# Pharmacokinetic characteristics of mesenchymal stem cells in translational challenges

**DOI:** 10.1038/s41392-024-01936-8

**Published:** 2024-09-13

**Authors:** Yunlong Shan, Mengying Zhang, Enxiang Tao, Jing Wang, Ning Wei, Yi Lu, Qing Liu, Kun Hao, Fang Zhou, Guangji Wang

**Affiliations:** 1 Key Laboratory of Drug Metabolism and Pharmacokinetics, Haihe Laboratory of Cell Ecosystem, State Key Laboratory of Natural Medicines, China Pharmaceutical University, Nanjing, China; 2 Jiangsu Renocell Biotech Co. Ltd., Nanjing, China

**Keywords:** Translational research, Stem-cell research

## Abstract

Over the past two decades, mesenchymal stem/stromal cell (MSC) therapy has made substantial strides, transitioning from experimental clinical applications to commercial products. MSC therapies hold considerable promise for treating refractory and critical conditions such as acute graft-versus-host disease, amyotrophic lateral sclerosis, and acute respiratory distress syndrome. Despite recent successes in clinical and commercial applications, MSC therapy still faces challenges when used as a commercial product. Current detection methods have limitations, leaving the dynamic biodistribution, persistence in injured tissues, and ultimate fate of MSCs in patients unclear. Clarifying the relationship between the pharmacokinetic characteristics of MSCs and their therapeutic effects is crucial for patient stratification and the formulation of precise therapeutic regimens. Moreover, the development of advanced imaging and tracking technologies is essential to address these clinical challenges. This review provides a comprehensive analysis of the kinetic properties, key regulatory molecules, different fates, and detection methods relevant to MSCs and discusses concerns in evaluating MSC druggability from the perspective of integrating pharmacokinetics and efficacy. A better understanding of these challenges could improve MSC clinical efficacy and speed up the introduction of MSC therapy products to the market.

## Introduction

Mesenchymal stem/stromal cell (MSC), which can be harvested and expanded from various adult and perinatal tissues, such as adipose tissue, bone marrow (BM), dental pulp, and umbilical cord (UC),^1^ exhibits diverse pharmacological effects, including immunomodulation, angiogenesis, and regenerative properties, rendering them promising for therapeutic applications.^2^ MSC therapy, which exerts the therapeutic potential of living cell preparations, has experienced explosive growth in both clinical deployment and expansion within the pharmaceutical marketplace for decades.^3,4^ Several MSC therapies approved by regulatory agencies and introduced to the commercial market have attracted increasing public attention. These include the successful approval of Prochymal (Remestemcel-L) in 2012 for treating graft-versus-host disease (GvHD) in Canada and Alofisel in 2018 for the treatment of Crohn’s disease in Europe (Table 1). The significant clinical benefits of MSC therapies are inspiring for further exploration and development.

**Table 1:** MSC products approved in different countries

Product	Origin	Therapeutic disease	Approved time	Approved region
Queencell	Autologous adipose tissue	Subcutaneous tissue injury	2010	Korea
Cellgram	Autologous BM	Acute myocardial infarction	2011	Korea
Cartistem	Allogeneic umbilical cord blood	Degenerative osteoarthrosis	2012	Korea
Cupistem	Autologous adipose tissue	Crohn’s Disease, Perianal fistulae	2012	Korea
Prochymal	Allogeneic BM	Graft-versus-host disease	2012	Canada, New Zealand
TEMCELL	Allogeneic BM	Graft-versus-host disease	2015	Japan
NeuroNata-R	Autologous BM	Amyotrophic lateral sclerosis	2014	Korea
Stempeucel	Allogeneic BM	Critical limb ischemia, Angiitis	2015, 2020	European Union, India
Stemirac	Autologous BM	Spinal cord injury	2018	Japan
Alofisel	Allogeneic adipose tissue	Crohn’s Disease, Perianal fistulae	2018, 2021	European Union, Japan

Many previous studies disconnected basic biomedical research and the potential druggability of MSCs. A limited understanding of the dynamic biodistribution and fate of MSCs within the body threatens the development of MSC therapies. The evaluation of the safety and efficacy of MSC therapy is limited by its unfavorable pharmacokinetic (PK) and pharmacodynamic (PD) properties.^5^ Pharmacokinetics, which describes how the body interacts with a drug, involves the processes of absorption, distribution, metabolism, and excretion, detailing the drug’s movement into, through, and out of the body over time.^6^ Unlike conventional drugs, MSCs can naturally migrate, localize, and even proliferate in specific tissues or compartments.^7^ In this review, we can consider the biodistribution of MSCs within the body as “distribution” and their fate as “metabolism and excretion”. Furthermore, MSCs are typically distinct from other cell therapies because their therapeutic efficacy depends not only on cell-to-cell contact but also on what is often referred to as a “hit-and-run” (or “touch and go”) mechanism—that is, through their rapid migration to damaged tissues and subsequent clearance following the release of paracrine effectors from their secretome, including soluble cytokines, growth factors, hormones, and miRNAs^8,9^ or the transfer of mitochondria to target cells through tunneling nanotubes,^10,11^ potentially leading to long-lasting effects.^12^ Thus, the lack of control over the localization, migration, and fate of MSCs is a translational challenge for MSC therapies.^13^

Numerous studies have shown that secretome-derived bioproducts containing bioactive molecules (such as proteins, lipids, and nucleic acids) and MSC-derived extracellular vesicles (EVs) retain the biological activity of parent MSCs and demonstrate similar therapeutic effects.^14^ The characteristics of secretome-derived bioproducts and MSC-derived exosomes have been comprehensively reviewed elsewhere.^15–17^ In this review, we first outline the key molecules involved in the motility of MSCs. We then provide a brief introduction to the various methods for tracking MSCs in vivo, highlighting their advantages and limitations. The core of the article focuses on the biodistribution and fate of MSCs in vivo, assessing the relationship between the kinetic characteristics and druggability of MSCs. Finally, we discuss the prospects of future opportunities for the clinical and commercial use of MSCs.

## The landscape in clinical MSC therapies

The success of clinical trials depends on understanding the mechanisms of drugs, but the clinical application of MSCs has frequently surpassed the understanding of their underlying mechanisms. A search using the keyword “mesenchymal stem/stromal cell” on ClinicalTrials.gov revealed more than 1600 related clinical trials, with more than 1500 clinical trials employing MSCs as a therapeutic intervention. With over 500 clinical trials of MSCs expected to be conducted by 2023, there is a wealth of information that can be leveraged to enhance our understanding of the factors influencing their successful implementation in human subjects (Table 2). These clinical trials are mostly in phase I, phase II, or combined phase I/II trials. Only a small percentage of trials are in phase III (comparing newer treatment approaches with standard or most well-known treatments) or phase II/III. Overall, MSCs appear to be well tolerated, with the majority of trials reporting no adverse reactions in the midterm. Only a few trials indicated mild and transient adverse reactions during the injection period. While MSCs exhibit a favorable safety profile, they often struggle to demonstrate significant efficacy in humans.^18^ Notably, no MSC therapies have received approval from the National Medical Products Administration (NMPA) of China or the U.S. Food and Drug Administration.

**Table 2:** Representative clinical trials of MSC therapies

General indication	Clinical indication	Cell source	Administration route	Clinical efficacy	Engineered	Phase	Trial number
Autoimmune diseases	Rheumatoid arthritis	Allogeneic mesenchymal precursor cells	Systemic	Yes	No	2	NCT03186417
	Systematic lupus erythematous	Allogeneic UC	Systemic	Promising	No	2	NCT03171194
Arthropathy	Osteoarthritis, knee	Autologous BM	Local	Yes	No	1/2	NCT01183728
	Osteoarthritis, knee	Autologous BM	Local	Yes	No	1/2	NCT01586312
	Osteoarthritis, knee	Autologous BM	Local	Yes	No	2	NCT02958267
	Focal cartilage lesions of the knee	Allogeneic BM	Local	Yes	No	1/2	NCT02037204
Blood disorder	Nonmalignant red blood cell disorders	Allogeneic BM	Systemic	No	No	2	NCT00957931
Cancer	Advanced gastrointestinal cancer	Autologous BM	Systemic	No	Yes	1/2	NCT02008539
	Metastases solid tumors	Autologous BM	Systemic	Unknown	Yes	1/2	NCT01844661
Cardiovascular diseases	Acute myocardial infarction	Allogeneic BM	Systemic	Yes	No	2	NCT00877903
	Chronic heart failure	Allogeneic mesenchymal precursor cells	Local	Yes	No	3	NCT02032004
	Heart failure	Autologous UC	Systemic	Yes	No	3	NCT05043610
	Ischemic stroke	Allogeneic BM	Systemic	No	No	2	NCT01436487
	Non-ischemic heart failure	Autologous BM	Systemic	No	No	2	NCT02467387
	Undergoing cardiac surgery	Autologous BM	Local	Yes	No	1/2	NCT00587990
	Cardiomyopathy due to anthracyclines	Allogeneic BM	Systemic	Yes	No	1	NCT02509156
	Chronic ischemic left ventricular dysfunction	Autologous BM	Local	No	No	1/2	NCT01087996
	Chronic ischemic left ventricular dysfunction	Autologous BM	Local	Yes	No	2	NCT02013674
	Nonischemic dilated cardiomyopathy	Autologous/Allogeneic BM	Systemic	Yes	No	1/2	NCT01392625
	Ventricular dysfunction, left	Autologous BM	Systemic	Yes	No	1/2	NCT00768066
	Dilated cardiomyopathy	Autologous BM	Systemic	Yes	No	2	NCT00629018
	Heart failure	Allogeneic BM	Local	Unknown	No	2	NCT00927784
Dental diseases	Periapical periodontitis	Allogeneic UC	Local	Yes	No	1/2	NCT03102879
Eye diseases	Advanced glaucoma	Autologous BM	Local	Unknown	No	1	NCT02330978
GvHD	Acute GvHD	Allogeneic BM	Local	Yes	No	1/2	NCT02379442
	Chronic GvHD	Allogeneic BM	Local	Unknown	No	2/3	NCT01526850
	Grade B to D acute GvHD	Allogeneic BM	Systemic	Yes	No	3	NCT02336230
	Grade B to D acute GvHD	Allogeneic BM	Systemic	Yes	No	3	NCT00366145
IBD	Crohn’s disease	Allogeneic adipose tissue	Local	Yes	No	3	NCT01541579
	Crohn’s disease	Allogeneic BM	Systemic	Yes	No	3	NCT00482092
	Ulcerative colitis	Allogeneic BM	Systemic	To be determined	No	2	NCT01240915
Kidney disorders	Acute kidney injury	Allogeneic BM	Systemic	No	No	2	NCT01602328
	Diabetic nephropathy	Allogeneic mesenchymal precursor cells	Systemic	Yes	No	1/2	NCT01843387
	Kidney failure	Allogeneic BM	Systemic	No	No	1/2	NCT01429038
	Renal transplantation	Autologous BM	Systemic	Yes	No	N/A	NCT00658073
Neurological disorders	Alzheimer’s disease	Allogeneic UC	Systemic	Unknown	No	1/2	NCT01547689
	Amyotrophic lateral sclerosis	Autologous BM	Local	Yes	Yes	2	NCT02017912
	Chronic progressive multiple sclerosis	Autologous BM	Local	To be determined	Yes	2	NCT03799718
	Diabetic peripheral neuropathy	Autologous BM	Systemic	Yes	No	N/A	NCT02387749
	Multiple sclerosis	Autologous BM	Systemic	To be determined	No	2	NCT02239393
	Spinal cord injury	Autologous BM	Local	No	No	1	NCT01909154
	Spinal cord injury	Allogeneic UC	Local	Yes	No	1/2	NCT02481440
	Spinal cord injury	Autologous BM	Local	Yes	No	1	NCT02165904
Respiratory disorders	Acute respiratory distress syndrome	Allogeneic BM	Systemic	N/A	No	1	NCT01775774
	Chronic obstructive pulmonary disease	Allogeneic BM	Systemic	No	No	2	NCT00683722
	Lung adenocarcinoma	Allogeneic UC	Systemic	To be determined	Yes	1/2	NCT03298763
	Acute respiratory distress syndrome	Allogeneic BM	Systemic	Yes	No	2	NCT02097641
	Idiopathic pulmonary fibrosis	Allogeneic UC	Systemic	Yes	No	1	NCT01385644
	Respiratory distress syndrome, adult	Autologous BM	Systemic	Unknown	No	2	NCT02112500
Skin disorder	Psoriasis vulgaris	Allogeneic UC	Systemic	Yes	No	1/2	NCT02491658

### Representative registered clinical trials of MSC therapies

Many completed clinical trials have demonstrated the efficacy of MSC infusion in treating a variety of diseases, such as GvHD, multiple sclerosis (MS), Crohn’s disease (CD), amyotrophic lateral sclerosis (ALS), myocardial infarction (MI), and acute respiratory distress syndrome (ARDS), among others (Table 2). Kabat et al. classified the indications for clinical trials into 14 groups, with those that could not be classified or had too few cases classified as “Other“.^19^ Among these clinical trials, MSC therapies for “neurological” diseases (17%) and “joint” diseases (15%) are the most common in registered indications. Cardiovascular disease (8.8%) and GvHD (8.3%) are closely related to this disease, with a higher percentage of phase III clinical trials. Only 84% explicitly specify the route of administration in MSC clinical trials.

### Clinical comparison of MSC therapy and cell-free therapy

Compared to MSC therapy, cell-free therapy utilizing the MSC secretome involves the delivery of multiple bioactive molecules rather than intact cells.^20^ Assessing the safety and efficacy of MSC-conditioned medium will be considerably less complicated.^21^ Furthermore, the production of conditioned medium is more economical, as it can be scaled up for mass production utilizing existing MSC populations under current good manufacturing practice conditions. Nonetheless, cell-free therapy products share similarities with conventional drugs in that they may necessitate prolonged administration and could result in the development of drug resistance and adverse reactions.^22^ However, cell therapy offers the distinct advantage of potentially conferring enduring effects with a single treatment that can last for several months, reducing the need for frequent medication and enhancing the resilience of the treatment regimen.^23^ Additionally, to enhance the efficacy of MSC therapy and their homing ability, the singular or combined application of bioengineering techniques shows promising potential to significantly overcome the current clinical challenges.

### Impact of different administration routes

The pharmacokinetics and biological properties of infused MSCs may be influenced by the route of administration.^13,24^ The administration routes in MSC clinical trials mainly include intravenous, intracardiac, intra-articular, intramuscular, intraosseous, intrathecal, intra-arterial, and implantation. Among the eight most frequently employed routes, intravenous infusion accounts for 43%.^19^ This preference for intravenous infusion is attributed to its ease of administration, low invasiveness, and high repeatability, making it the most frequently utilized method in treatment.^25^

Local administration is commonly employed because it allows direct delivery to the disease site. Forty-nine percent of registered MSC clinical trials utilized local delivery by 2018.^26^ Many MSC therapies are locally administered for various diseases, including lumbar pain, anal fistula, and chronic heart failure, in late-stage clinical trials.^27^ Local administration of MSCs is a more controllable approach, making it easier to access disease sites and often yielding better therapeutic responses.^28^ However, in certain cases, less than 5% of the administered cells have been observed to remain at the injection site just hours after transplantation.^29^ Moreover, local administration of MSCs also results in secondary distribution but is limited to locally vascularized areas such as the heart.^30^ The poor persistence of MSCs after local administration is due to several factors, such as cell death from the inhospitable microenvironment at the disease site and ineffective integration into the tissue.^31^ Compared to systemic administration, direct injection of MSCs into damaged tissue sites lacks systemic homing processes, but the persistence of MSCs in target tissues remains similar to that of systemic administration.

Systemic administration of MSCs is more minimally invasive and can avoid tissue calcification issues compared with local injection.^32^ Among the approved MSC products, treatments for GvHD and spinal cord injury are administered systemically. Although many studies in animals and humans have shown that intravenously infused MSCs tend to stick to the lungs,^33^ systemic administration of MSC therapy has been used in most clinical trials by 2023. Due to nonspecific uptake by blood vessels in nontarget tissues, higher doses are needed for MSCs to achieve effective treatment levels in target organs and tissues. The administration of 10^5^–10^7^ MSCs per kilogram is required based on patient weight,^34,35^ leading to widespread biodistribution in tissues and organs such as the lung, liver, and gastrointestinal tract.^36^ An increasing number of MSC clinical trials are using systemic administration, highlighting the need for a comprehensive understanding of the pharmacokinetics of MSCs in the body. The detailed process of systemic administration of MSCs in vivo can be found in the next section.

## Molecules of governing MSC motile potency during homing

**Fig. 1:**
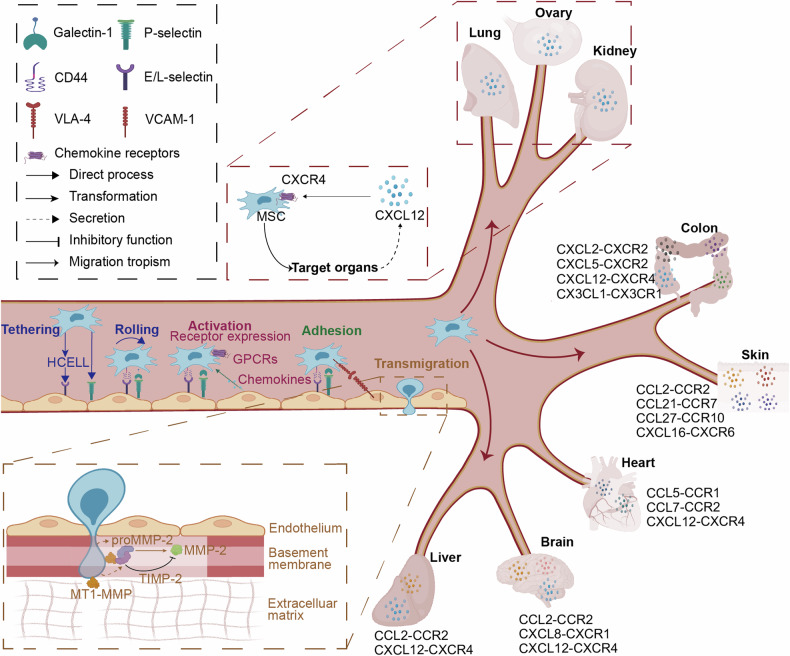
Multiple steps in mesenchymal stem cells (MSCs) homing. The homing processes of MSCs are dependent on a series of molecules of motile potency, including selectins, integrins, chemokine receptors, and proteases. Selectins, which are expressed on endothelial cells (ECs), capture MSCs by interacting with their ligands and then mediating the rolling of MSCs. MSCs can thus be activated by exposure to chemokines, consequently leading to integrin-dependent firm adhesion to the endothelium. At the appropriate location, MSCs cross the endothelial barrier by secreting different proteases before continuing chemokine-mediated migration

After systemic administration, MSCs enter the blood circulation and cross the vascular endothelium at lesion sites through four steps: (1) tethering and rolling, (2) activation, (3) adhesion, and (4) transmigration (Fig. 1). This process involves various proteins that regulate MSC motility and interactions with endothelial cells (ECs). The ligands expressed on the surface of MSCs mediate their rolling action on the vascular wall upon binding to selectins. The G protein-coupled chemokine receptors on MSCs receive activation signals, leading to integrin-dependent adhesion to the endothelium. Finally, MSCs secrete proteases to break down the EC barrier, completing the homing process.^7,37,38^

### Selectin ligands

The homing process is initiated through tethering events mediated by selectin ligands on MSCs and selectins on ECs (Table 3), with MSCs rolling after attaching to ECs.^39^ Hematopoietic cell E-selectin/L-selectin ligand (HCELL), a functional form of CD44 containing an sLex-like epitope, binds to E-selectin/L-selectin and functions as a marker of MSC motility.^40,41^ P-selectin also plays a role in mediating the rolling of MSCs through interacting with P-selectin glycoprotein ligand (PSGL-1),^42^ and blocking P-selectin decreases the binding of MSCs to ECs.^39^ Suila et al. reported that Galectin-1, rather than PSGL-1, tends to serve as a marker of MSC motility, as MSCs preferentially interact with P-selectin on ECs through Galectin-1, which is independent of the sLex epitope.^43^ The complex interplay of various selectin ligands is crucial for the efficient adhesion of MSCs to ECs.

**Table 3:** Molecules governing MSC motility during homing

Molecule	Ligand/Receptor/Substrate	Ligand/Receptor source	Function	MSC organism	Cell source	Strategies to improve MSCs homing	Homing target	Ref.
Galectin-1	P-selectin	Endothelium	Tethering and rolling	Human	Umbilical cord blood	Enzymatic modification (pronase)	Injury area	^43,333^
CD44 (α-1,3-gamma glycosylation)	E-selectin	Endothelium; Ischemic kidney	Shear-resistant adhesion	Human; Mouse	Adipose; BM	Enzymatic modification (fucosyltransferases)	Inflammatory sites; Kidney	^41,334–336^
	L-selectin	Endothelium		Human	Adipose; BM	Enzymatic modification (fucosyltransferases)	BM	^41,334,335^
VLA-4	VCAM-1	Endothelium	Firm adhesion and rolling	Human	BM; Placentas	Priming (LLP2A)	BM; Fetal tissue	^40,46,337^
CCR1	CCL2	Hippocampus	Homing to the cortices and hippocampus	Human	Nasal olfactory mucosae	Genetic modification (overexpression: lentivirus)	Myocardial infarction area	^65^
	CCL5	Infarct adjacent border zone area of the heart	Homing to the heart infraction sites	Mouse	BM	Genetic modification (overexpression: lentivirus)	/	^63^
		Granulation tissue of the ear	Homing to the ear wound sites	Human	Periodontal ligament	Genetic modification (overexpression: Kaposi’s sarcoma-associated herpesvirus)	Inflamed ears	^73^
	CCL7	Urethral	Homing to the muscle bundles in the urethra	Rat	BM	Genetic modification (overexpression: lentivirus)	CCL7 injection site	^64^
CCR2	CCL2	Skin wound tissues	Homing to the skin wound sites	Human	UC	Genetic modification (overexpression: lentivirus)	Injured skin tissues	^67^
		Liver	Homing to the liver lesions	Human	UC	Genetic modification (overexpression: lentivirus)	Injured liver	^68^
		Hemisphere	Homing to hemisphere ischemic sites	Human	BM	Genetic modification (overexpression: lentivirus)	Ischemic hemisphere	^69^
		CD133^+^ glioblastoma stem cells	Homing to the brain	Human	UC	Genetic modification (overexpression: lentivirus)	Brain injury	^70^
		Hippocampus	Homing to the cortices and hippocampus	Human	Nasal olfactory mucosae	Genetic modification (overexpression: lentivirus)	Lesioned hippocampus	^65^
	CCL7	Cardiac fibroblasts	Homing to the heart infraction sites	Rat	BM	Genetic modification (overexpression: plasmid)	Injured myocardium	^71^
CCR3	CCL5	Injured and inflammatory sites	Homing to the wound sites	Human	Periodontal ligament	Genetic modification (overexpression: Kaposi’s sarcoma-associated herpesvirus)	Injured and inflammatory sites	^73^
CCR4	CCL2	Astroglia cell	Homing to the hemisphere lesions	Human	BM	Priming (polyethylenimine)	Brain injury	^74^
CCR5	CCL5	Glioma-associated microglia	Homing to the retina	Mouse	BM	Genetic modification (overexpression: lentivirus)	Degenerating retina	^96^
CCR6	CCL20	Epithelial cells	Homing to skin	Mouse	BM	Genetic modification (overexpression: plasmid)	Skin wounds	^75,338^
CCR7	CCL21	Keratinocytes	Homing to the skin wound sites	Mouse	BM	Priming (culture media additives)	Wounds	^78^
		High endothelial venules of secondary lymphoid organs	Homing to the secondary lymphoid organs	Mouse	BM	Genetic modification (Overexpression: plasmid)	Secondary lymphoid organs	^77^
		Lung	Homing to the lung	Rat	BM	Genetic modification (overexpression: lentivirus	Injured lung	^339^
CCR9	CCL25	Thymic dendritic cells	Homing to the thymic	Mouse	BM	Genetic modification (overexpression: lentivirus)	Thymus	^80,340^
CCR10	CCL2	Hippocampus	Homing to the cortices and hippocampus	Human	Nasal olfactory mucosae	Genetic modification (overexpression: lentivirus)	Lesioned hippocampus	^65^
	CCL27	Epidermis, dermis	Homing to the interfollicular dermis	Mouse	BM	Genetic modification (overexpression: plasmid)	Normal skin	^82^
CXCR1	CXCL8	Glioma cells	Homing to the hemisphere	Human	Umbilical cord blood	Genetic modification (overexpression: plasmid)	Gliomas	^85^
CXCR2	CXCL2	Colon	Homing to the colon inflamed sites	Human	BM	Genetic modification (overexpression: mRNA transfection)	Inflammatory bowel	^87^
		Tongue	Homing to the inflamed mucosa of the tongue	Human	BM	Genetic modification (overexpression: lentivirus)	Radiation-induced oral mucositis	^88^
	CXCL5	Colon	Homing to the colon inflamed sites	Human	BM	Genetic modification (overexpression: mRNA transfection)	Inflammatory bowel	^87^
CXCR3	CXCL10	Ear	Homing to the ear inflamed sites	Human	BM	Genetic modification (overexpression: lentivirus)	Inflamed lesions	^341^
CXCR4	CXCL12	Brain	Homing to the brain	Human	UC; BM	Genetic modification (overexpression: lentivirus; adenovirus)	Brain injury	^70,130^
		Ovary	Homing to the ovary	Human	Amnion	/	Injured ovaries	^92^
		BM	Homing to the cortical and trabecular bone	Rat	BM; Adipose	Genetic modification (overexpression: adenovirus)	BM	^342,343^
		Osteoblast	Homing to the fracture sites	Rat	BM	Short-Wave	Fracture site	^344^
		Liver	Homing to the liver ischemic sites	Human	BM	Hypoxia	Injured liver	^345^
		Liver	Homing to the liver ischemic sites	Human	UC	Priming (rapamycin))	Injured liver	^170^
		Heart	Homing to the heart infraction sites	Rat	BM	Genetic modification (overexpression: lentivirus)	Heart of ischemia/perfusion injury	^146^
		Lung	Homing to the alveoli	Rat	BM	Genetic modification (overexpression: lentivirus)	Injured lung	^346^
		Lung epithelial cells	Homing to the lung	Human	BM	Genetic modification (overexpression: lentivirus)	Alveolar epithelial cells	^91^
		Lung metastatic tumor cells	Homing to the lung	Rat	BM	Genetic modification (overexpression: plasmid)	Lung tumors	^347^
		Intestinal tissue	Homing to the intestine inflamed sites	Mouse	BM	Genetic modification (overexpression: lentivirus); priming (IL-1β)	Intestine	^138,140^
		Kidney	Homing to the glomeruli and tubulointerstitium	Rat	BM	Hypoxia	Ischemic acute kidney	^348^
		Renal tubules cells	Homing to the kidney ischemic sites	Mouse	BM	Hypoxia	Renal ischemia/reperfusion	^349^
		Basal layer of the epidermis, hair follicles	Homing to the epidermis and hair follicles of wounds	Mouse	BM	/	Burn wounds	^350^
CXCR5	CXCL13	Ear	Homing to the ear inflamed sites	Human	BM	Genetic modification (overexpression: lentivirus)	Inflamed ears	^97^
CXCR6	CXCL16	Skin wound tissues	Homing to the skin wound sites	Mouse	BM	Genetic modification (overexpression: plasmid)	Skin wounds	^75^
CX3CR1	CX3CL1	Intestinal epithelial cells	Homing to the colon	Rat	BM	Genetic modification (overexpression: lentivirus)	Inflammatory bowel	^99^
MMP-1	Collagen, gelatin, laminin	/	Breakdown the endothelial basement membrane	Human	BM	Priming (IL-1β; IL-6)	Damaged tissues	^106,112^
MMP-2	Collagen, gelatin, laminin	/	Breakdown the endothelial basement membrane	Human	BM	Priming (TGFβ; IL-1β; TNFα; IL-6)	Inflammatory sites	^106,109,112^
MMP-13	Collagen, gelatin, laminin	/	Breakdown the endothelial basement membrane	Human	BM	Priming (IL-1β; IL-6)	Inflammatory sites	^106,112^
MT1-MMP	Collagen fibrils	/	Breakdown the interstitial stroma	Human	BM	Priming (TGFβ; IL-1β; TNFα; PDGF)	Inflammatory sites	^106,107,109^
TIMP-1	MMPs	/	Inhibit MMPs	Human	BM	Genetic modification (knock down: siRNA)	Inflammatory sites	^106,109,112,351^
TIMP-2	MMPs	/	Inhibit MMPs; MMP-2 proenzyme activation	Human	BM	Antibody conjugation (TIMP antibodies)	Damaged tissues	^106,109,112,351^
TIMP-3	MMPs	/	Inhibit MMPs	Human	BM	Antibody conjugation (TIMP antibodies)	Inflammatory sites	^104^
TIMP-4	MMPs	/	Inhibit MMPs	Human	BM	Antibody conjugation (TIMP antibodies)	Damaged tissues	^112^
Sialyl Lewis X	P-selectin	/	Promote rolling response	Human	BM	Protein conjugation (Sialyl Lewis X)	Inflamed tissues	^352^
P-selectin binding peptide	P-selectin	/	Enhance their targeting to vascular injury sites	Rat	Adipose	Protein conjunction (Peptides: DAEWVDVS)	Vascular injury sites	^353^
Peptides	Cysteine-rich protein 2	/	Improving the homing efficiency	Human	BM	Protein-conjunction (Peptides: CRPPR)	Myocardial infarction	^151^

### Integrins

Integrins are transmembrane receptors that act as interfaces between extracellular and intracellular compartments and facilitate cell-extracellular matrix (ECM) adhesion.^44^ MSCs express a broad spectrum of integrins, including β1, β2, β3, β5, α1, α2, α3, α4, α5, α6, α7, α8, α9, αX, αV, and αD^45^ (Table 3). The integrin VLA-4 (α4β1), which is composed of CD49d (α4) and CD29 (β1), is highly expressed on human MSCs^46^ and plays a crucial role in the adhesion and rolling of MSCs. Vascular cell adhesion molecule-1 (VCAM-1) is an immobilized protein expressed on ECs that can bind to CD29, resulting in fast-rolling interactions under appropriate shear stress.^47^ Blocking CD29 or VCAM-1 abolishes MSC rolling, indicating that these rolling interactions depend on adhesion. This subsequently diminishes MSC migration to the injured liver and ischemic myocardium.^40^ Compared to that of CD29, the amount of CD49d on MSCs is insufficient, which may limit the homing ability of MSCs.^48^ Overexpression of CD49d enhances transendothelial migration in vitro and increases homing to the BM in vivo,^49^ demonstrating that VLA-4 is a suitable marker for MSC motility. Notably, MSCs also express intercellular cell adhesion molecule (ICAM-1, the ligand for αLβ2) naturally, but the significance of ICAM-1 in MSC homing still needs to be clarified. Integrin, a significant molecule involved in MSC motility, can generate actin-based forces to pull the cell body forward through more stable interactions between MSCs and ECs.

### Chemokine receptor family

Chemokines and their receptors are now recognized as important mediators of MSC homing. Human chemokines are a superfamily of 48 ligands that bind to 19 different G protein-coupled chemokine receptors, regulating cell proliferation, differentiation, chemotaxis, and other physiological processes.^50^ They can be categorized into several groups depending on their functions.^51,52^ Inflammatory chemokines are upregulated during inflammation, promoting leukocyte recruitment and migration toward injury or infection sites.^53^ Homeostatic chemokines regulate adaptive immune responses and contribute to stem cell systemic migration.^51^ MSC activation is triggered by G protein-coupled chemokine receptors that respond to inflammatory signals, with chemokines binding to chemokine receptors on MSCs, leading to integrin recruitment and increased adhesiveness.^54^ In addition, the increased integrin affinity is attributed to rapid lateral mobility triggered by chemokines.^55^ MSCs express a broad panel of chemokine receptors, including CCR1-7, CCR9-10, CXCR1-6, and CX3CR1,^56–60^ but the expression of most these receptors is low and decreases after in vitro expansion, which limits studies on MSC homing and therapeutic efficacy.^61^ The chemokine-chemokine receptor network also plays significant roles in the tropism of MSCs from the bloodstream toward different organs or tissues in response to inflammation or injury (Table 3).

#### CCR1

CCR1 on MSCs is a key marker of motile potency, with a broad spectrum of chemokines acting as natural ligands. CCL3, which is produced by lymphocytes, monocytes, and macrophages in response to proinflammatory agents/cytokines, can accelerate MSC migration by interacting with CCR1 on MSCs.^62^ Stimulating CCR1-overexpressing MSCs with CCL5 increased the ability of MSCs to home to injured sites and restored cardiac function.^63^ CCL7, which is significantly expressed in the urethras of rats during stimulated birth injury, is a ligand for CCR1, and pretreatment combined with CCR1-MSC overexpression increases MSC engraftment to the mid-urethra.^64^ The lesioned hippocampus expresses increased CCL2, which recruits human olfactory ectomesenchymal stem cells (OE-MSCs) in vitro.^65^ CCL2 may bind to and activate CCR1 and CCR10 in OE-MSCs through an unknown migration pathway.

#### CCR2

Previous studies have shown that CCL2 (the ligand of CCR2) is widely present in certain inflammatory diseases and monocyte recruitment in injured tissue mainly depends on CCR2.^66^ Therefore, CCR2 on MSCs may also play an important role in migration. CCL2 induces MSC migration by activating CCR2 in vitro, and the CCL2-CCR2 axis promotes the homing of MSCs to various organs or tissues, including the heart, liver, brain, skin, and tumor.^67–71^ Huang et al. showed that CCR2 overexpression on MSCs enhanced their homing to the ischemic hemisphere and improved therapeutic outcomes.^69^ Moreover, CCR2 also plays a crucial role in regulating the motility of MSCs and promoting their recruitment to liver injury sites.^72^ CCL7, another ligand for CCR2, could induce MSCs to migrate toward the injured myocardium, resulting in beneficial remodeling in the infarct zone.^71^

#### CCR3 and CCR4

MSCs typically do not express CCR3, but following infection with Kaposi’s sarcoma-associated herpesvirus (KSHV), they exhibit increased CCR3 expression and enhanced migration ability in vitro. Knockdown of CCR3 or blockade of its ligand CCL5 can partially suppress MSC migration, suggesting that the CCL5-CCR3 axis may promote MSC homing.^73^ Treatment with the cationic molecule polyethylenimine (PEI) dose-dependently increased CCR4 expression on MSCs, and the brain homing efficiency of MSCs significantly increased after intravenous administration of PEI in a rat model of brain injury.^74^

#### CCR6 and CCR7

Overexpressing CCR6 enhances MSC migration to skin wound sites.^75^ BM-MSCs expressing CCR6 can transmigrate across ECs in response to CCL20.^76^ Transfected MSCs with enhanced CCR7 expression showed improved migration to secondary lymphoid organs in response to SLC/CCL21, leading to prolonged survival in GvHD mice.^77^ Additionally, injection of CCL21 into the periphery of wounded skin significantly increased MSC recruitment to wound sites and accelerated wound closure in skin wound models.^78^

#### CCR9 and CCR10

CCL25 acts as a chemoattractant for various immune cells and is a ligand for CCR9.^79^ CCL25 has been found to mediate the migration of MSCs expressing different levels of CCR9 in vitro.^80^ In addition, CCL27 (the ligand of CCR10), a skin-specific chemokine, is predominantly expressed by keratinocytes in the epidermis of skin (injured/uninjured/exudates), and its upregulation recruits MSCs to migrate toward wounded skin.^81^ In vivo studies have shown that systemically administered MSCs overexpressing CCR10 migrate to the skin in response to CCL27.^82^

#### CXCR1 and CXCR2

CXCL8 (also known as interleukin 8, IL-8) is an inflammatory chemokine that can interact with CXCR1 and CXCR2.^83^ Ringe et al. found that MSCs migrate to zones with high concentrations of CXCL8 in a dose-dependent manner through in vitro chemotaxis assays.^83^ This process was observed in gliomas, in which CXCL8 was identified as an inducer of human umbilical cord blood-derived MSC migration toward glioma cells both in vitro and in vivo.^84,85^

CXCR2, a molecule with various ligands, is crucial for leukocyte migration toward inflamed BM sites.^86^ Genetic modification can upregulate CXCR2 expression on MSCs, enhancing their mobilization and accelerating healing.^87,88^ CXCL7 recruits human BM-MSCs in vitro and may influence their migration through the IL-8 receptor signaling pathway. The activation of chemokines that stimulate CXCR1 and CXCR2 suggests a synergistic mechanism for CXCL7-driven MSC migration.^89^

#### CXCR4

CXCL12 (SDF-1)-CXCR4 has already been the most widely studied MSC chemotactic axis.^90^ CXCL12 is secreted from various tissues, including the tumor, intestine, skin, ovary, bone marrow, liver, lung, kidney, and heart, and has been confirmed to recruit intravenously injected MSCs expressing CXCR4.^70,91,92^ Recent studies have investigated various methods for enhancing the migration and therapeutic efficacy of MSCs by increasing CXCR4 expression, such as hypoxia, chemical compounds, or cytokines, during MSC expansion in vitro.^93^ Notably, hypoxic pretreatment has been found to increase CXCR4 expression, promote migration, and reduce the apoptosis of MSCs in vitro.^94^ In addition to its role in migration and survival, the CXCL12-CXCR4 axis is also critical for MSC cytokine secretion.^95^

#### Others

MSCs overexpressing CCR5 exhibit enhanced migration following transplantation into the degenerating retina.^96^ In mice with allergic contact dermatitis, CXCL10 and CXCL13 mRNA levels were upregulated in inflamed ears. The number of MSCs overexpressing CXCR3 or CXCR5 increased in these ears, indicating that the CXCL10-CXCR3 and CXCL13-CXCR5 axes enhance MSC migration.^97^ Transwell assays revealed that CXCR6 is involved in the transmigration of MSCs across the EC layer in response to CXCL9.^76^ Exposure of MSCs to hypoxia increased CX3CR1 expression and migration in vitro.^98^ Intravenous injections of CX3CR1-overexpressing MSCs led to the generation of long-lasting MSCs in the inflamed colon of mice, even more so than in the liver and lung.^99^

### Matrix metalloproteinase family

Traversing the protein fibers of the ECM, which is present in all tissue types, is crucial for cells to reach target sites in circulation. Basement membranes separate the epithelium or endothelium from the stroma.^100^ MSCs break down basement membranes by secreting matrix metalloproteinases (MMPs), which are involved in various physiological and pathological processes because of their capacity to degrade ECM components.^101,102^ MMPs can also function as motility markers of MSCs, influencing their homing efficiency (Table 3). Gelatinases, MMP-2, and MMP-9 are major contributors to the breakdown of basement membranes.^103^ However, MSCs secrete MMP-2 but not MMP-9, identifying MMP-2 as the key player involved in MSC transmigration and invasion.^101^ Knocking down MMP-2 significantly reduced the transendothelial migration ability of these cells.^104^ Chemerin, a chemoattractant produced by the liver, adipocytes, and lung, manipulates the transmigration of intravascularly administered MSCs by upregulating MMP-2 expression.^105^ MT1-MMP, a membrane-tethered metalloenzyme, not only regulates MSC trafficking through the interstitial ECM both in vitro and in vivo but also directs MSC differentiation programs.^106^ PDGF-BB stimulates MSC migration and proliferation, upregulating MT1-MMP expression on MSCs.^107^

The expression of four tissue inhibitors of metalloproteinases (TIMPs), which inhibit the active form of all MMPs, is induced by inflammatory cytokines or chemokines.^108^ Silencing of TIMP-1 enhanced MSC migration, indicating that both TIMP-1 and TIMP-2 play opposing roles in the homing process.^109^ Additionally, TIMP-1 activity is responsible for the antiangiogenic effects of MSCs in inflamed lymph nodes of mice.^110^ TIMP-3, produced by MSCs, has been recognized as a soluble factor that positively impacts EC function in a mouse model of traumatic brain injury.^111^ TIMP-4 has been found to be expressed at very low levels in BM-MSCs.^112^

### Strategies to improve MSC homing

The success of MSC therapies is facilitated by the effective delivery of living cells to injured tissues to carry out their biological functions. Beyond the exclusive use of anticoagulants,^26^ various bioengineering strategies (Table 3), including magnetic guidance, radiotherapeutic techniques, genetic modification, enzymatic modification, protein/antibody conjugation, and priming, have been extensively developed and applied to enhance MSC homing efficiency.^27,38^ Genetic modification methods, such as viral or mRNA transfection to transiently or permanently overexpress specific molecules governing MSC motility, have been discussed in the previous sections.

Unlike genetic modifications, cell surface engineering aims to chemically alter the surface of MSCs directly through enzymatic modification and protein/antibody conjugation, resulting in temporary but effective enhancements in homing. One such strategy is the enzymatic modification of CD44, which converts CD44 to HCELL through sugar modifications, thereby enabling MSCs to home to the BM via E-selectin and L-selectin.^113^ This modification also increases MSC infiltration into pancreatic islets threefold following intravenous administration and durably reverses hyperglycemia.^114^ Attaching anti-ICAM-1 or anti-VCAM-1 antibodies to MSCs has been shown to improve retention and homing to target tissues.^115,116^ The conjugation of E-selectin-binding peptides onto MSC membranes is utilized to manipulate cell-microenvironment interactions, particularly for targeting cell delivery to specific tissues.^117^ Gundlach et al. developed a bispecific antibody designed to bind CD90 on MSCs and myosin light chain 1 on ischemic myocardium.^118^

Priming MSCs with small molecules or soluble factors is a simple and effective strategy to boost the molecules that regulate MSC motility. The upregulation of CXCR4 and MMP-9 in MSCs treated with valproic acid and lithium results in enhanced MSC homing, improved functional recovery, and reduced infarct volume in the brains of rats in a cerebral ischemia model.^119^ In addition, individual MSCs were encapsulated in hydrogels or microgels to increase the residence time of MSCs in target tissues. Mao et al. demonstrated that encapsulating MSCs in alginate-poly-d-lysine-alginate microgels significantly improved the delivery efficiency of MSCs to target tissues. Magnetic labeling of MSCs has been explored as a strategy for targeted tissue delivery.^120^ The enhanced homing ability of MSCs labeled with iron oxide was observed to be ten times greater when these cells were intravenously infused into a rat model. One week post-injection, magnetic MSCs demonstrated improved penetration into both the inner and outer retina in comparison to nonmagnetic MSCs.^121^

Understanding the roles of various molecules expressed or secreted by MSCs is crucial for MSC engineering. Bioengineering strategies not only improve the efficacy of MSCs but also accelerate their accumulation in target tissues. These strategies can enhance clinical outcomes by overcoming the challenges of limited MSC persistence and inadequate homing to targeted sites.

## Target organs of MSC homing after systemic administration

The localization of MSCs within various organs post-administration provides critical insights into their interactions with tissues and target cells, which are pivotal for the efficacy of MSC-based therapies.^122^ MSCs have the capacity to home to inflammatory or injured sites, and their biodistribution varies due to their diverse pathogenesis in different disease models, where chemokine–chemokine receptor axes play an important role (Fig. 1).

### Brain

Although MSCs can cross the blood‒brain barrier (BBB), no signal from cells can be detected by any of the tracking modalities in the healthy brain after intravenous injection.^123^ The biodistribution of MSCs in injured brains is completely different from that in healthy brains. MSCs migrate to injured brains at 4 h and reside in the brain until day 11, which is the endpoint of detection after intravenous administration.^124^ The homing efficiency and retention time are diverse due to the application of different tracking techniques and administration routes. Oshra et al. monitored the biodistribution of MSCs by a nanoparticle-based computed tomography (CT) imaging technique after orthotopic injection. Gold nanoparticle-labeled MSCs could be detected at 1 h, 24 h, and one month post-injection. However, another study showed that no signal from MSCs can be tracked only on day 2 after intra-arterial transplantation.^125^ After using mannitol to enhance the penetrability of the BBB, MSCs could be detected on day 7 and day 28 after infusion compared with those in the control group without mannitol pretreatment.^126^

The conventional SDF-1/CXCR4 and CCL2/CCR2 chemokine axes can participate in the homing of MSCs to injured brains as well as other organs.^127–129^ To improve the homing efficiency of MSCs to injured brains, various strategies can be employed for the modification of MSCs prior to administration. The overexpression of CXCR4 or CCR2 in MSCs is used mostly to increase the targeting ability of cells after intracerebroventricular injection and carotid injection.^69,130^ Shahror et al. demonstrated that MSCs overexpressing growth factor 21 exhibit enhanced homing efficiency in the damaged brain, thereby improving the therapeutic effects on traumatic brain injury.^131^ Inflammatory cytokines can also improve the homing ability of MSCs. For example, the upregulation of IL-8 in injured brains has been confirmed, which has been shown to improve the homing ability of BM-derived stem cells.^132^ Furthermore, pretreatment of MSCs with other soluble factors and chemical agents can also enhance their ability to home to injured brains. Valproate and lithium have been shown to boost the accumulation of MSCs in the tissue of injured brains.^119^ Other factors, such as IL-3, IL-6, IL-1β, and IFNγ, can also promote the migration of MSCs to injured tissues.^133^ In addition to gene modification and chemical pretreatment, physical modification can improve the therapeutic efficacy of MSCs by enhancing the ability of cells to home to the brain. More polycluster superparamagnetic iron oxide nanoparticle-labeled MSCs accumulate in the injured brain after intravenous injection and have longer retention times in the brain than control MSCs.^134^

### Colon

Numerous studies have shown that MSCs accumulate preferentially in the lungs and do not migrate to the colon after intravenous injection in a normal mouse model.^135^ However, the homing of MSCs to damaged colons was increased in a dextran sulfate sodium-induced colitis model.^136^ MSCs were monitored by in vivo optical bioluminescence imaging at 48 h after administration, and imaging revealed an increased number of MSCs in the inflamed colon in colitis mice (1.5–5.5%) in comparison with that in healthy mice (0.3–1%).^137^

The SDF-1/CXCR4 axis, CXCL2/CXCR2 axis, and CXCL5/CXCR2 axis can play critical roles in the homing of MSCs to the injured colon.^87,138^ Another study revealed that upregulating CX3CL1 in injured colon tissue and transducing MSCs with lentivirus expressing CX3CR1 markedly enhanced cell accumulation at injured colon sites after 2 h, 24 h, and 8 days intravenous injection.^99^ Although more MSCs home to damaged colon sites in disease models than in healthy models, the homing efficiency remains insufficient to achieve optimal therapeutic effects. A series of modification strategies could be employed to improve the migration of MSCs to the inflamed colon. Fu et al. constructed dual-functionalized MSCs that overexpress CX3CR1 and IL-25, which can promote the delivery of MSCs to inflamed colons and improve therapeutic effects on inflammatory bowel disease.^99^ Among the chemokine receptors, CXCR2 plays an important role in regulating the migration of MSCs to damaged tissues, and transient CXCR2 expression on MSCs can enhance the migration of cells to damaged colons.^87^ When coupled with an anti-VCAM-1 antibody, MSCs had greater migration efficiency than control MSCs and completely attenuated colitis.^139^ The expression of CXCR4 can be upregulated by pretreating MSCs with IL-1β, which can enhance the migration rate of cells to the colon.^140^ In addition to IL-1β, IFNγ can also increase the homing of MSCs to injured tissues of the colon.^141^ The upregulation of ICAM-1 or CXCR4 demonstrated a positive regulatory relationship with the homing efficiency of MSCs to the injured colon.^142^ Chemical methods for improving the targeting ability of MSCs have been utilized in many studies. Polyribocytidylic acid, the ligand of Toll-like receptor (TLR3), has been confirmed to promote MSC homing by activating TLR3.^143^

### Heart

Intravenously administered MSCs can be detected in infarcted hearts on days 0.5, 1, 2, 4, 8, and 16, peaking on day 1 and then decreasing with time.^144^ Another study revealed that less than 1% of administered MSCs accumulate in hearts under normal conditions; however, more than 3% of intravenously injected cells could still be monitored in infarcted hearts after 3 months.^145^ Andrzejewska et al. also demonstrated that MSCs can be tracked in injured heart regions for up to 7 days following intravenous injection.^60^

Many reports have shown that chemokines, such as SDF-1, CCL7, CCL2, CCL5, CCL25, and CX3CL1, are upregulated in infarcted hearts and are involved in the homing of MSCs to these damaged areas.^146–149^ Overexpression of protein kinase C ε and monocyte chemotactic protein-3 (MCP-3 or CCL7) can improve both the capacity of MSCs to migrate to infarcted hearts and their therapeutic effects.^71,150^ In addition, coupling MSCs with a palmitated peptide resulted in an increased number of MSCs in ischemic heart tissue compared with noncoated controls.^151^

### Kidney

Only 5.4% of administered MSCs migrated to healthy kidneys after intravenous injection, but treatment with cis-platinum increased the release of chemokines from injured kidneys, enhancing MSC homing to 6.4%.^152^ Furthermore, the difference in the distribution of MSCs is partly attributed to the route of administration, with more MSCs accumulating in injured kidneys 15 min after intra-arterial injection than after intravenous injection in a mouse acute kidney injury model. Most MSCs disappeared within 2 days, with only a barely detectable positive signal remaining in both groups.^153,154^ Another study reported that more MSCs could be detected in injured kidneys than in normal kidneys 2 h after tail vein injection^155^; these MSCs exhibited longer retention times and primarily accumulated in the glomeruli of the injured kidneys.^156^

Emerging data show that the CCL2/CCR2 axis and SDF-1/CXCR4 axis participate in the migration of MSCs to the kidney.^32,157^ The therapeutic efficacy of MSCs is closely associated with the number of MSCs migrating to the injured region of the kidney. Many methods for enhancing the homing efficiency of MSCs have been reported. CXCR4 overexpression significantly increased the migratory capacity of MSCs in injured kidneys and has been used in many disease models.^158^ When coupled with kidney injury molecule-1, MSCs migrate to ischemic kidneys more and reside in kidneys for a longer time.^159^ Pretreating MSCs with erythropoietin, the transforming growth factor β, and insulin-like growth factor-1 can improve the homing ability and restore the function of injured kidneys.^160–162^ Additionally, ultrasound can enhance the homing capacity of MSCs through the use of various nanoparticles in acute kidney injury.^163,164^

### Liver

Compared to the normal model, the injured liver model exhibited greater homing efficiency (28.7–35.3%).^165^ Furthermore, in a normal mouse model, no MSCs were detectable at 5 days after intravenous injection.^122^ However, MSCs were detected in CCl_4_-injured livers 24 h post-injection,^166^ with the greatest homing observed in the injured liver tissues 1 week after intravenous injection.^165,167^ Furthermore, compared to intravenous injection, hepatic artery delivery shows greater homing efficiency (20–30%) to injured livers.^168^

The use of inflammatory cytokines, hypoxia, chemical agents, and genetic modifications could improve the homing of MSCs to injured livers. Cytokines play a critical role in the homing of MSCs to injured livers. The upregulation of cytokines such as stem cell factor-1, hepatocyte growth factor (HGF), MMPs, and chemokines in injured livers accelerates their homing to the liver.^169^ A previous study revealed that the upregulation of CCL2 in damaged livers and the overexpression of its receptor CCR2 in MSCs can enhance the delivery of MSCs to damaged livers.^68,170^ Transfecting MSCs with lentivirus overexpressing c-Met can improve the survival rate of rats in an acute liver failure model by enhancing the homing capacity of MSCs.^171^ Activation of the HGF/c-Met signaling pathway can improve the migration of MSCs to injured sites, so overexpression of HGF is desirable for the targeting of damaged liver sites by MSCs.^172^ Pretreating MSCs with IFNγ can increase the homing ability of MSCs to damaged liver tissues.^173^ IL-6 can also improve the homing capacity of MSCs in the fibrotic liver.^174^ In addition, pretreatment of MSCs with chemical agents such as sodium nitroprusside, preoperative resveratrol, and melatonin accelerates MSC homing, thereby improving therapeutic efficacy through increased localization of MSCs in the injured liver.^175–177^

### Lung

MSCs are initially trapped in the lungs after intravenous injection and then redistributed to other organs with high blood flow, such as the liver, primarily because the structural characteristics of the lung microvasculature cause the majority of MSCs to become sequestered there.^33^ However, the population of MSCs residing in the lungs diminishes due to increased permeability under injured conditions in the initial stage of injection. Emerging data confirm that transplanted MSCs home more effectively to injured lungs than to normal lungs, as chemokines released from inflammatory sites in the blood system recruit these cells to injured areas.^178–180^

The regulation of signaling pathways could similarly enhance the homing capacity to injury sites. MSCs with downregulated Hippo signaling, which can promote the migration of cells, attenuate lung injury in ARDS mice.^181^ With the knockout of vimentin, fewer MSCs colonize injured lungs.^182^ After transduction with a lentiviral vector carrying the E-prostanoid 2, the capacity of MSCs to home to injured lung tissues improved significantly.^183^ Pretreating MSCs with granulocyte colony-stimulating factor can promote the ability of MSCs to home to injured lungs by upregulating the expression of CXCR4.^184^ Furthermore, the retention time of injected MSCs in mice could be increased via radiation.^185^

### Skin

MSCs are not detectable in healthy mouse skin after intravenous injection. However, increased numbers of MSCs can be detected in injured skin, and this process is mediated by multiple chemokine axes. Alexeev et al. demonstrated the upregulation of CCL27 in wounded skin tissue and the importance of the CCL27/CCR10 axis in the homing of MSCs to damaged skin.^82^ Increased expression of CXCR6 on MSCs enhanced the accumulation of cells in wound sites on the skin, which was assisted by the upregulation of the ligand CXCL16 in damaged skin.^75^ In addition, the ability of the CCL2/CCR2 axis and CCL27/CCR10 axis to enhance the homing efficiency of MSCs has been confirmed in several studies.^67,78,186^ After pretreatment with inflammatory cytokines, such as IL-17, and plant-derived components, such as protocatechuic acid, cannabinoids, and icariin, a sufficient number of MSCs can migrate to wounded skin.^187,188^ Junction adhesion molecule A may accelerate the repair of wounded skin and improve the therapeutic effects of MSCs by increasing their ability to migrate to damaged skin.^189^

### Other organs

The target organs of MSCs also include the ear and ovary. MSCs can be tracked at 24 h after transplantation in the injured ovary.^190^ Another study showed that MSCs were located in the interstitium of the ovary at 4 weeks after tail vein injection.^191^ The SDF-1/CXCR4 axis participates in the homing of MSCs to the ovary.^92^ Hyaluronic acid improves the homing ability of MSCs to the sites of injured ears.^192^ Moreover, the glycoengineering of MSCs significantly enhances their homing to tumors.^193^

Overall, after peripheral intravenous injection, MSCs are initially trapped in the lungs,^33^ with a smaller portion passing through to reach organs with rich blood flow, such as the liver,^165^ kidney,^152^ and brain,^123^ and minimal numbers of MSCs migrating to the skin.^82^ Most in vivo distribution studies have been conducted in mice or rats, and given the significant differences in vascular diameters between these animals and humans, these results cannot be directly translated to humans. MSCs labeled with ^111^In-oxine show a gradual decrease in lung retention and an increase in liver and spleen retention over time in humans.^194^ These findings suggest that MSCs may display unconventional pharmacokinetic characteristics, with the levels of inflammatory cytokines in vivo and molecules regulating MSC motility being crucial parameters for pharmacokinetic evaluation.

## The fate of MSC after transplantation

**Fig. 2:**
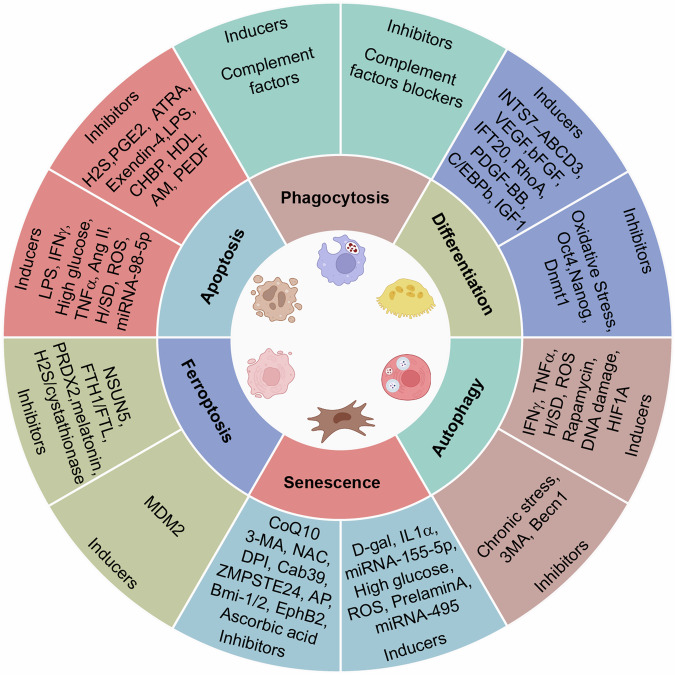
Regulators and effects of different MSC fates. Regulated by a variety of intrinsic or extrinsic factors, MSCs develop into different destinies. MSCs have stem cell properties and diverse differentiation directions under distinct conditions. Phagocytosis of MSCs is mediated mostly by the monocyte‒macrophage system. Autophagy and senescence can be activated in ROS-enriched environments or under other conditions. Many MSCs undergo apoptosis. Changes in the functions and phenotypes of MSCs and their consequent effects can be detected when MSCs progress to different fates

Several preclinical MSC therapy studies have demonstrated that MSCs do not persist in the body long-term and are gradually cleared over time.^27,195^ There are significant differences in the persistence of MSCs in vivo across various experiments. Some studies indicate that MSCs can survive in the body for only 24–28 h,^33,196^ whereas other studies have shown that MSCs are still detectable up to 120 days post-administration.^197^ The long-term persistence of MSCs in vivo carries the risk of tumor formation, while short-term persistence may restrict their therapeutic efficacy. Hence, understanding the fate of MSCs is essential for assessing their safety and efficacy. While metabolism and excretion represent the fate of conventional drugs, apoptosis, autophagy, differentiation, ferroptosis, phagocytosis, and senescence are the fates of MSCs (Fig. 2). An increasing understanding of the fate of MSCs after intravenous injection has advanced further research into the mechanisms underlying their therapeutic effects.

### Apoptosis

Apoptosis, a programmed cell death process, is mediated by diverse signaling pathways and is triggered by multiple factors, such as cellular stress, DNA damage, and other proapoptotic agents.^198^ MSCs undergo apoptosis in different target organs after administration, whether in a physiological or pathological environment. He et al. investigated the apoptosis of MSCs after infusion in a mouse liver and spinal cord injury model and attributed the therapeutic effects to the release of phosphatidylserine from apoptotic cells. Apoptotic MSCs were first detected at 2 h post-injection in the liver and lung and markedly decreased at 12 h and 24 h, respectively.^72^ Another study revealed that in a GvHD model, MSC apoptosis was induced by perforin released from cytotoxic cells one hour after injection, and the MSCs were primarily localized in the lung and spleen. Subsequently, the phagocytosis of apoptotic MSCs by macrophages is essential for the immunosuppressive effects of MSCs, as it leads to the production of indoleamine 2,3-dioxygenase.^8^ However, Swee et al. reported that MSC apoptosis occurs not only in a pathological environment but also in a physiological state after infusion. After intravenous injection, MSCs rapidly undergo apoptosis in the lungs of recipient mice. Furthermore, the results suggested that apoptosis is not dependent on host cytotoxic or alloreactive cells.^199^

The apoptosis of MSCs, a complex process influenced by various factors, is driven by inflammatory cytokines such as IFNγ and TNFα, which induce the upregulation of inducible nitric oxide synthase and the production of nitric oxide through the activation of the STAT1 signaling pathway.^200,201^ MSCs have been utilized in the treatment of conditions such as ischemia and myocardial infarction, where the proapoptotic effects are primarily orchestrated through the intricate PI3K/AKT signaling cascade due to low oxygen levels and a lack of essential nutrients in the blood.^202–205^ The overexpression of certain microRNAs under pathological conditions can initiate apoptosis by downregulating antiapoptotic protein expression, as demonstrated by a study in which miRNA-98-5p triggered apoptosis in MSCs by targeting insulin-like growth factor type 2 mRNA-binding protein 1 and modulating the PI3K/AKT and p53 signaling pathways.^206^ Increasing evidence indicates that enhanced reactive oxygen species (ROS) generation at injury sites, along with high glucose and other contents released from red blood cells, can induce MSC apoptosis.^207–211^

### Autophagy

Autophagy plays an important role in maintaining the therapeutic effects of MSCs.^212^ As one of the intracellular degradation systems, autophagy is a complex process composed of a variety of signaling pathways that can be triggered by diverse inducers.^213^ Among the diverse fates of MSCs, autophagy can be activated by various factors, such as cell starvation, inflammation, and oxidative stress.^214^ MSCs were detected in the lungs at 24 and 48 h post-administration, with a significant increase in the autophagy marker microtubule-associated protein 1 light chain 3 (MAP1LC3). However, MSCs were not detected in the spinal cord until 72 h post-injection, at which time they could still be detected 120 h later, accompanied by a significant increase in MAP1LC3.^215^ A reduction in IGF-1 expression results in the downregulation of the AKT/mTOR signaling pathway and the upregulation of autophagy in aged MSCs exposed to hypoxic conditions.^216^ Autophagy also enhances MSC survival in vitro under conditions of starvation, hypoxia, or exposure to ROS.^217–219^

### Differentiation

MSCs, a heterogeneous subset of multipotent stromal stem cells, can differentiate into adipocytes, osteocytes, and chondrocytes. Emerging studies have shown that MSCs can also differentiate into ECs and neurons at injury sites after transplantation.^12^ Therefore, the differentiation potential of MSCs is vital for their therapeutic effects. Some MSCs express EC-specific markers after intracerebral administration in a rat model of stroke, revealing that the increase in vascular density is mediated by the differentiation of MSCs into ECs.^220^ Another study revealed that human adult dental pulp MSCs preferentially differentiated into astrocytes after intracerebral transplantation in a focal cerebral ischemia model.^221^ Only a few MSCs differentiate into other cells, which mainly occur in the bone, brain, and blood vessels after administration via different pathways.^222^

The differentiation of MSCs is regulated by various signaling pathways that intricately coordinate this complex process. Intraflagellar transport 20 plays a critical role in determining MSC fate, and its depletion results in a marked increase in adipocyte differentiation.^223^ In contrast, the suppression of sex-determining region Y box 9 protein hinders the adipocyte differentiation of MSCs through the inhibition of the transcription factor CCAAT enhancer binding protein β.^224^ The interaction between integrator complex subunit 7 and ATP-binding cassette subfamily D member 3, along with the presence of serum IGF-1, facilitates the osteoblast differentiation of MSCs, offering potential for treating osteoporosis.^225,226^

### Ferroptosis

Ferroptosis, which was originally described by Dixon et al. in 2012, represents a distinct form of regulated cell death that is mechanistically different from apoptosis.^227^ This iron-dependent process is characterized by the accumulation of intracellular iron and increased levels of lipid peroxidation.^228,229^ Ferroptosis is inhibited by the overexpression of NOP2/Sun RNA methyltransferase 5 or ferritin heavy chain/light-chain in MSCs.^230^ Conversely, the murine double minute 2-TLR4 axis and the prominin2-BACH1 axis can also participate in ferroptosis in MSCs.^231,232^ The occurrence of ferroptosis in MSCs after transplantation reduces their efficacy, and intervention strategies are urgently needed to improve their therapeutic effect. Chen et al. demonstrated that increased levels of peroxiredoxin-2 in MSCs can improve therapeutic outcomes in a neurogenic erectile dysfunction rat model by suppressing ferroptosis.^233^ Strategically modulating metabolic pathways to inhibit ferroptosis in MSCs can substantially increase the therapeutic potential for ameliorating hepatic damage.^234–236^

### Phagocytosis

MSCs have been widely recognized as promising tools for treating autoimmune diseases because of their ability to cross-talk with various immune cells. Several experiments revealed that nearly half of MSCs were subjected to phagocytosis after intravenous injection.^237^ MSCs are usually trapped in the lungs and disappear within a day under normal conditions. The fate of MSCs after their disappearance from the lungs was elucidated, showing that MSCs were primarily phagocytized by blood-derived monocytes in the lungs and subsequently redistributed to the liver.^195^ Another study revealed that MSCs are injured upon contact with blood compounds due to the complement system,^238,239^ and this apoptosis and injury facilitate their phagocytosis by monocytes.^8^ In addition to monocytes, tissue-resident macrophages can also phagocytize intravenously injected MSCs. In a mouse model of asthma, MSCs were primarily localized in the alveolar and capillary walls 1 h post-injection and were phagocytized by lung-resident macrophages 24 h post-injection.^240^ Furthermore, MSCs can be phagocytized by splenic macrophages at 3 h and 24 h after intravenous injection in tumor-bearing mice.^241^

### Senescence

MSCs tend to become senescent after injection due to crosstalk with the microenvironment, which hinders their clinical application and leads to undesirable therapeutic effects. MSCs become senescent with increased expression of senescence-associated genes during long-term in vitro culture and due to replicative senescence and their niche after in vivo administration.^242^ The mechanisms and induction factors of senescence have been described in many studies. ROS not only induce autophagy but also promote the senescence of MSCs.^243,244^ D-galactose (D-gal) can promote intracellular ROS generation, which markedly induces cell senescence. Coenzyme Q10 and ascorbic acid inhibit the pro-senescence effect of D-gal on MSCs through AKT/mTOR signaling.^244,245^ In addition to ROS, microRNAs can induce cellular senescence, and inhibiting microRNA-45 and microRNA-155-5p can rejuvenate senescent MSCs.^246,247^ Overexpression of apelin and Erb-B2 receptor tyrosine kinase 4 could rejuvenate senescent MSCs via the PI3K/AKT and AMPK signaling pathways after administration and enhance the therapeutic efficacy of MSCs in a myocardial infarction model.^248,249^ Knockdown of CD26 reduces senescence-associated cytokine secretion and potentiates the therapeutic effects of MSCs by delaying the senescence of MSCs in a mouse emphysema model.^250^

Premature senescence of MSCs, which occurs more frequently during the pre-processing and early post-injection stages than natural senescence, remains a significant hurdle in clinical application.^251^ Multiple signaling pathways are involved in the regulation of the premature senescence of MSCs, with the ROS/mTOR/4EBP1/p70S6K1/2 pathway and the FoxO3a pathway playing key roles in this process.^251,252^ Many strategies have been employed in studies to prevent premature senescence as much as possible and improve the therapeutic efficacy of MSCs. Walnut kernel oil and defatted extracts enhance MSC stemness and delay senescence.^253^ Depletion of GATA binding protein 4 can hinder SASP-dependent senescence in MSCs by suppressing the NF-κB pathway.^254^

Currently, the apoptosis, autophagy, ferroptosis, and other fates of MSCs have been investigated in vitro. However, many findings on the in vivo fate of MSCs after injection are unknown, which limits the detection and evaluation of MSC efficacy to an extent. In the future, elucidating the fate of MSCs within various target organs in vivo will be crucial, necessitating the development of new probes and biotechnology for such investigations.

## The imaging and tracking modalities of MSC

**Fig. 3:**
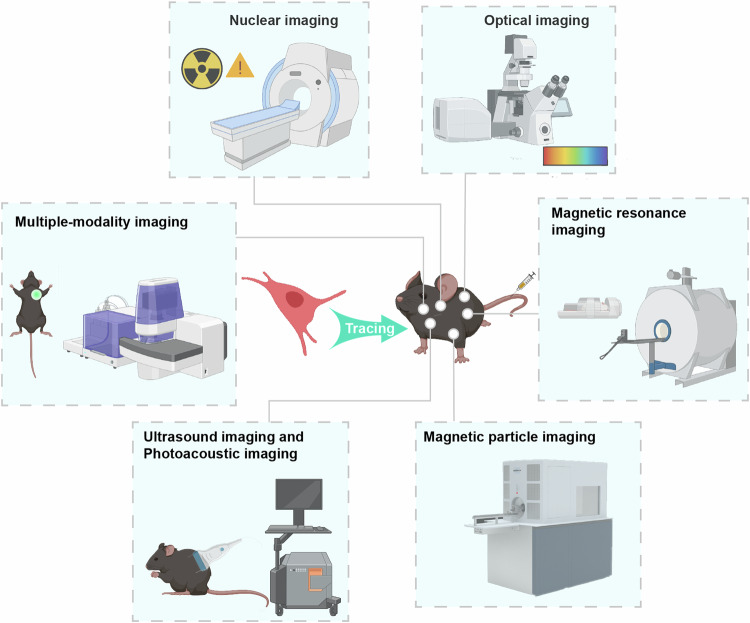
Multimodal tracing strategies for MSCs in vivo. Precise and effective detection methods are vital for exploring the migration and distribution of mesenchymal stem cells (MSCs). Currently, there are various detection techniques available. Nuclear imaging tracks the biodistribution of cells by imaging radiolabeled tracers. Optical imaging, including bioluminescence imaging (BLI) and fluorescence imaging (FLI), plays an important role in researching the biodistribution of MSCs in different disease models. Cell tracking with magnetic resonance imaging (MRI) requires labeling cells with contrast agents. Magnetic particle imaging (MPI) can detect nanoparticle tracers at any time and location in the body with greater spatial and temporal resolution than MRI. Ultrasound imaging and photoacoustic imaging are noninvasive and emerging imaging modalities. The development of multiple-modality imaging is an emerging trend in MSC tracking and clinical research

The homing and persistence of transplanted MSCs in vivo remain poorly understood due to technical limitations, which hinders their clinical translation. Precise and effective detection methods are vital for exploring the migration and distribution of MSCs. Current methods for imaging and tracking in vivo include gene quantitative detection, optical imaging, magnetic resonance imaging (MRI), nuclear medicine imaging, ultrasound imaging, and photoacoustic imaging (Fig. 3),^255^ each of which has advantages and disadvantages (Table 4).^256^ To precisely and effectively detect the biodistribution of MSCs in vivo, a combination of different approaches is required.

**Table 4:** Comparison of technologies for detecting the distribution of MSCs^256^

Technology	Clinical application	In vivo*/*In vitro	Single-cell resolution	The lower limit of detection (Number of cells)	Advantages	Disadvantages
MRI	Yes	In vivo	No	10^4^	Noninvasiveness; High spatial resolution; Whole body scan	Low sensitivity; Unquantifiable
PET/SPECT	Yes	In vivo	No	10^4^	Noninvasiveness; High sensitivity; Whole body scan; Quantifiable	Low spatial resolution; Short half-life
BLI	No	In vivo	No	10^3^	Noninvasiveness; High sensitivity; Long-term detection; Distinguish live/dead cells	Limit on detection depth; No single-cell resolution
FLI	No	In vivo	Yes	10^3^	Distinguish live/dead cells; Single-cell resolution	Invasiveness; Limit on detection depth, High operation requirement
Flow cytometry	No	In vitro	Yes	10^3^	Distinguish live/dead cells	Multiple antibodies label; Low precision; Low stability
Q-PCR	Yes	In vitro	No	10^2^	High sensitivity; High specificity; Wide application; Label-free	Unable to distinguish living/dead cells; Limited detection area
IHC	Yes	In vitro	Yes	10^2^	High sensitivity; High specificity; Single-cell resolution	Limited detection area; Selective imaging of specific protein; No dynamic detection

### Methods for detecting MSC pharmacokinetics

Flow cytometry, immunohistochemistry (IHC), and real-time quantitative polymerase chain reaction (Q-PCR) detection systems are commonly used for the invasive detection of MSCs.^257,258^ With convenient operation and high sensitivity, Q-PCR can detect and quantify MSCs quickly without labeling, but it cannot distinguish between living and dead cells.^259^ Flow cytometry is performed by collecting limited tissues for analysis and requires labeling with specific antibodies, which is less accurate and stable than Q-PCR.^260^ Although IHC can directly detect the distribution of MSCs, it cannot achieve long-term in vivo tracking of cells.^261^

In vivo tracking of MSCs involves direct and indirect labeling, commonly using radioisotopes, magnetic nanoparticles, and fluorescent dyes for direct labeling. After incubation with the above labeling materials and administration to animals, MSCs can be detected by positron emission tomography (PET)/single-photon emission computed tomography (SPECT), MRI, and in vivo fluorescence imaging. These tracking technologies can noninvasively and continuously monitor the dynamic distribution of MSCs in preclinical and clinical trials.^256^ In addition, superparamagnetic iron oxide nanoparticles are used most often in MRI technology,^262^ and the radioisotopes ^64^Cu-PSTM, ^18^F-FDG, ^111^In-oxine, and ^99^mTc are the primary tracer agents used in PET/SPECT.^263^ Although the direct labeling method is more convenient, markers are diluted through cell division and shed from cells over time, reducing the accuracy of long-term tracking.

Indirectly labeled MSCs are tracked by detecting fluorescent proteins or specific proteins combined with a nuclide probe, which are transferred into cells by gene modification. Optical imaging technologies, classified as fluorescence imaging and bioluminescence, are popular for tracking living cells. Fluorescence imaging (FLI) detects cells by fluorescence microscopy and mainly monitors the distribution of cells in animals because of limits on detection depth.^264^ Bioluminescence-based imaging (BLI) is a minimally invasive technique for experimental animals in which luciferase is continuously and stably expressed in MSCs.^265^

### Optical imaging

Based on the detection of light emission from molecules after excitation via an internal charged-coupled device camera, optical imaging, which mainly consists of BLI and FLI, plays an important role in researching the biodistribution of MSCs in different disease models.^266^ With high accessibility and the possibility of long-term tracking, optical imaging is widely used for tracking MSCs. Compared to nuclear imaging modalities, optical imaging has fewer negative effects on cell proliferation, viability, and differentiation. Furthermore, most of the labeling reagents for optical imaging are suitable for longitudinal detection.^267^

FLI relies on various fluorophores that can be excited by specific wavelength light sources, with the emitted light collected by fluorescence signaling components and transformed into images by signal detection and amplification components.^268^ The exogenous labeling of MSCs using fluorescence has been recognized as an important imaging approach in vivo with excellent accessibility and operability. One of the conventional exogenous fluorescent dyes is hloromethyl-dialkylcarbocyanine, which is frequently used to track labeled cells, especially those that bind to the cell membrane.^269^ In addition, quantum dots are nanomaterials with intense fluorescence and diverse colors that can directly label MSCs, offering advantages over conventional fluorescent agents.^270^ To enable long-term cell tracking without affecting cell proliferation and potency, novel fluorescent probes are essential. Liu et al. designed a biocompatible and photostable nanoprobe made of polycaprolactone and di(thiophene-2-yl)-diketopyrrolopyrrole that retained strong fluorescence for up to 4 weeks for MSC differentiation tracing.^271^ However, one drawback of labeling MSCs directly with fluorescent reagents is a false positive signal. When transfected with fluorescent protein plasmids or virus vectors, MSCs have a greater signal-to-noise ratio than when directly labeled with fluorescent agents. The biodistribution of MSCs labeled with green fluorescent protein has been tracked, and the action of these cells post-administration have been characterized.^272,273^ Compared with other fluorescent reagents, near-infrared (NIR) fluorescent dyes with emission wavelengths ranging from 740 nm to 1700 nm exhibit reduced tissue absorbance, scattering, and autofluorescence, thereby enabling greater depth of penetration. After being labeled with NIR, MSCs can be continuously traced in vivo for up to 21 days, and liver regeneration can be visualized in an acute liver failure model.^274^

BLI relies on the photon emission generated by the catalytic conversion of luciferase, which facilitates the intramolecular oxidation of luciferin in the presence of ATP and oxygen within luciferase-expressing MSCs.^275^ All of the studies tracked cells in real time after injection for up to 4 weeks, which is superior to nuclear imaging.^276–278^ BLI can also avoid interference from background and biological autofluorescence signals during the imaging process.

In summary, optical imaging is widely used for MSC tracking due to its long-term tracking ability, lower cytotoxicity compared to nuclear imaging, diverse fluorescent reagents, and accessibility. However, its poor tissue penetrability and light scattering limit its use in small animal imaging (Table 5).

**Table 5:** Methods for tracking MSCs

Imaging modality	Spatial resolution	Species and tissue of MSCs	Observation time	Labeling methods and agents	Labeling time	Imaging purposes	Imaging sites	Experimental model	Ref.
BLI	3–5000 μm	hUC-MSC	7 days	Lentiviral vectors	7 days	Biodistribution tracking	Whole body	Normal	^196^
		hAD-MSC	/	CMV vector	3 days	Real-time tracking of stem cell viability, proliferation, and differentiation	Whole body	Normal	^354^
		hBM-MSC	4 weeks	Adenoviral vectors	2 days	Distribution of MSCs at tumor sites	Tumor	Tumor-bearing mice	^276^
		Rat BM-MSC	4 weeks	Lentiviral vectors	7 days	Cell survival evaluation in defect sites	Calvaria defect site	Calvarial critical sized defect model	^278^
FLI	1 mm	human embryonic stem cells-derived M-MSCs	28 days	Lentiviral vectors	4 days	Interaction of MSCs with the vessel system of the bladder	Bladder	Interstitial cystitis/bladder pain syndrome	^273^
		human embryonic stem cells-derived M-MSCs	6 weeks	Lentiviral vectors	/	Monitor the action of multipotent stem cells in real time	Bladder	Interstitial cystitis/bladder pain syndrome	^355^
MRI	10–100 μm	hBM-MSC	24 h	Amino-polyvinyl alcohol coated SPIONs	4 h	Biodistribution of MSCs after injection	Whole body	Normal	^292^
		hUC-MSC	10 days	Ferumoxytol	3 days	MSCs spheroids tracking	Whole body	Normal	^356^
		Rat BM-MSC	2 days	Perfluorocarbon	24 h	Biodistribution of MSCs after lung injection	Whole body	Normal	^290^
PAI	10–100 μm	Murine BM-MSCs	3 days	Citrate-coated prussian blue particles	1, 3, 6, 12, 24, and 48 h	Provide a new noninvasive and high resolution approach to image traumatic brain injury, monitor the recovery process, and especially trace MSCs	Brain	Brain injury	^296^
		MSCs	10 days	Inert gold nanorods coated with IR775c, a reactive oxygen species sensitive near-infrared dye	6 h	Longitudinal tracking of MSC viability with a high spatial and temporal resolution	Heart	Normal	^297^
		hBM-MSC	4.6 days	Iron-oxide nanoparticle	24 h	Monitor the transplantation, biodistribution, and clearance of MSCs	Whole body	Normal	^295^
PET	1-2 mm	hUC-MSC	7 days	^89^Zr-oxine	20 min	Biodistribution of MSCs expressing anticancer protein TRAIL	Tumor	Lung mesothelioma	^285^
		hBM-MSC	1.5 h	2-deoxy-2-[^18^F]fluoro-D-glucose ([^18^F]FDG)	1 h	Biodistribution of MSCs in a different animal model *via* different routes of administration	Whole body	Normal	^284^
SPECT	1–2 mm	hBM-MSC	24 h	^99^mTc-HMPAO	15 min	Biodistribution of MSCs	Whole body	Normal	^282^
		ADS1-hMSCs	21 days	A novel gold nanoparticle (GNP)coated with glucose	3 h	Track the migration and exact localization of GNP-labeled hMSCs	Brain	Depression	^357^
BLI + FLI		hBM-MSC	4 weeks	Lentiviral vectors	3 days	Biodistribution tracking	BM	Normal	^272^
				BLI: lentiviral vectors/FLI: magnetic nanoparticles (MNP) conjugated with fluorophores	2 days/18 h	Action analysis and tracking of MSCs	Brain	Stroke	^277^
BLI + MRI		Rat BM-MSC	10 days	pCMV-Luciferase2-mKate2- PAI/SPION	2 days	Biodistribution of MSCs after injection	Liver	Acute liver failure	^358^
		Murine BM-MSCs	2 days	Lentiviral vectors; superparamagnetic iron oxide nanoparticles	5 days;24 h	The viability, whole-body biodistribution, and intrarenal biodistribution of MSCs	Whole body and kidney	Mouse renal injury	^154^
FLI + SPECT		hBM-MSC	21 days	Au-Albumin-indocyanine green-poly-L-lysine (AA@ICG@PLL NP)	24 h	Tracking of MSCs	Lung	Lung fibrosis	^359^
FLI + PAI		hUC-MSC	21 days	Near-infrared II fluorescent dye-modified melanin nanoparticles (MNP-PEG-H2)	4 h	Biodistribution of MSCs in liver	Liver	Acute liver failure	^274^
MPI + MRI		Murine BM-MSCs	6 h	Ferucarbotran; perfluoropolyether	4 h	Assess the cellular sensitivity of MPI and 19 F MRI for detection of MSCs	Whole body	Normal	^294^
PAI + SPECT		Rat BM-MSC	7 days	A cobalt protoporphyrin IX-loaded mesoporous silica nanoparticle (CPMSN) into a^125^I-conjugated/spermine-modified dextran polymer (^125^I-SD)	8 h	Real-time tracking of MSCs with high spatial resolution	Brain	Transient middle cerebral artery occlusion	^299^
BLI + FLI + PET		Murine BM-MSCs	27 days	Adenoviral vectors	3 days	Biodistribution of MSCs in different inflammatory pathologies	Whole body	Diabetes; injury/wound healing; tumor-bearing mice	^298^
FLI + MRI + SPECT		Rat BM-MSC	14 days	^125^I-fSiO4@SPIOs	1 h	Biodistribution of MSCs after injection	Brain	Transient middle cerebral artery occlusion	^360^
FLI + PAI + PTT		hUC-MSC	5 days	Mesoporous silica-coated gold nanostars integrated with indocyanine green	24 h	Biodistribution of MSCs in tumor	Tumor	Breast cancer	^361^

### Nuclear imaging

Nuclear imaging tracks the biodistribution of MSCs by imaging tracers that have been labeled with radionuclides. There are three nuclear imaging techniques: PET, SPECT, and planar gamma scintigraphy.^279^ PET and SPECT will be summarized in this review since they are used more widely than planar gamma scintigraphy in the tracking of MSCs.

The gamma-ray photons with well-defined energy levels emitted from SPECT radioactive tracers can be captured directly by a gamma camera, where they are eventually digitized to provide 3D information on the biodistribution of MSCs^279^ Although the SPECT imaging modality offers relatively high sensitivity and quantifiability, its widespread application in the long-term tracking of MSCs is limited due to the use of radiotracers with short half-lives. ^99m^Tc, which has a half-life of only 6 h, can be detected persistently for 3 days in vivo after injection.^280^ Compared with ^99m^Tc, ^111^In-labeled MSCs can be detected up to 14 days after intravenous injection because of their long half-life (*t*_1/2_ = 2.81 days). Another drawback of SPECT is its cytotoxicity associated with the use of radiotracers. Although it has no impact on the viability or differentiation of human MSCs labeled with ^111^In, many other experiments have shown that radiotracer labeling can affect MSC viability and differentiation in a dose-dependent manner.^281^ Owing to the imaging properties of SPECT, the signal of MSCs in deep tissues could also be captured by a detection camera. When human BM-MSCs were labeled with ^99m^Tc, their biodistribution in arthrosis was fully depicted using a gamma camera.^282^

PET imaging involves capturing the signals of gamma photons emitted in opposite directions by positron-emitting radionuclides using a ring detector, followed by data processing and image reconstruction. In contrast to SPECT, the PET imaging modality has higher spatial and temporal resolution but shorter cell tracking time in vivo. However, similar to SPECT, PET imaging is limited by time-dependent ionizing radiation injury to target cells and the short half-life of tracers, which restricts its use in long-term cell tracking.^279^ The isotopic tracers commonly used in PET imaging are ^18^F-fluorodeoxyglucose (FDG) and ^18^F-FHBG.^270,283^ Nose et al. tracked the biodistribution of MSCs in multiple animal species using PET imaging with ^18^F- FDG following various routes of administration.^284^ While ^89^Zr, which has a longer half-life (*t*_1/2_ = 78.4 h) than ^18^F (*t*_1/2_ = 109.7 min), can also be used for tracking MSCs, Patrick et al. labeled MSCs with ^89^Zr to show its delivery to the lungs in a mouse lung cancer model up to 7 days post-injection.^285^ While nuclear imaging offers good tissue penetrability and sensitivity, its limited spatial resolution and short imaging time make it unsuitable for precise and longitudinal MSC imaging (Table 5).

### Magnetic resonance imaging

MRI is a powerful imaging tool with superior spatial resolution and tissue penetrability under preclinical and clinical conditions.^286^ This modality can also provide 3D information on MSC biodistribution as well as nuclear imaging. Tracing cells with MRI requires labeling cells with contrast reagents, such as superparamagnetic iron oxide nanoparticles (SPIONs), paramagnetic metals (Gd^3+^ and Mn^2+/3+^) or micron-sized iron oxide particles.^287–289^ While ferumoxide-labeled MSCs were observed only in the lung, perfluorocarbon-labeled MSCs dispersed to other distant organs.^290^ Most cell tracking studies use dextran or carboxy dextran-coated SPIONs, which are designed for uptake by phagocytic cells^291^; however, their impact on MSC proliferation and function must be evaluated. Novel SPIONs coated with amino-polyvinyl alcohol, which offer greater colloidal stability, greater solubility, greater biocompatibility, and lower toxicity, have been developed and are primarily taken up by MSCs.^292^

Despite its high spatial resolution and tissue penetrability, MRI has low sensitivity and long imaging times, making it difficult to track labeled cells throughout the whole body and determine their migration beyond damaged tissue. Furthermore, the accessibility of MRI is undesirable due to the high cost of imaging instruments (Table 5).

### Magnetic particle imaging

Magnetic particle imaging (MPI), which usually involves the use of noncytotoxic SPIONs, is a relatively new tomographic imaging technique (Table 5). MPI can detect nanoparticle tracers at any time and space in the body with greater spatial and temporal resolution than MRI.^293^ Sehl et al. compared the sensitivity of MPI and MRI using perfluoropolyether-labeled MSCs and revealed that MPI reliably detected 4000 MSCs, while MRI detected 256,000 MSCs at the same time.^294^ Zheng et al. utilized MPI to dynamically track and quantify the biodistribution of SPION-labeled MSCs following intravenous injection in mice. One hour post-injection, MSCs primarily accumulate in the lungs before gradually migrating to the liver within 1 day.^295^

### Photoacoustic imaging and ultrasound imaging

As a non-invasive and emerging imaging modality, photoacoustic imaging (PAI) offers rich optical contrast, high ultrasonic resolution, and fast data acquisition, making it suitable for real-time imaging (Table 5). Given these advantages, Li et al. demonstrated that Prussian blue particle-labeled MSCs were able to cross the BBB and subsequently migrate to injured brain regions.^296^ Dhada et al. developed a contrast agent with inert gold nanorods coated with IR775c, a ROS-sensitive near-infrared dye, enabling the measurement of MSC viability and longitudinal location by combining ultrasound imaging with PAI.^297^

### Multiple-modality imaging

With the development of MSC clinical research, real-time, low cytotoxicity, high temporal and spatial resolution, and long-term imaging modalities are required (Table 5). Since a single imaging modality cannot simultaneously meet all these requirements, multiple-modality imaging has emerged in recent years.^33^ Combining nuclear imaging with bioluminescence-based imaging enables long-term cell tracking and is complemented by anatomical information from CT, effectively offsetting the limitations of each method.^298^ Ultrasound imaging and PAI offer high contrast, flexibility, and fast acquisition for real-time MSC transplantation guidance, while MRI with anatomical imaging resolution and SPECT with high sensitivity and quantitative capability are ideal for long-term monitoring of cell biodistribution and migration in vivo. Yao et al. combined these modalities to monitor engrafted MSCs in real-time and track their long-term biodistribution.^299^ The optical-MRI modality can improve the sensitivity of MSC detection in vivo and the resolution of imaging.^255^

In recent years, in vivo tracking imaging technologies have developed rapidly. Compared with single detection technology, employing multiple methods provides more comprehensive monitoring of MSC distribution in vivo. By combining Q-PCR with various imaging techniques, the dynamic spatial distribution of living cells, along with anatomical structures and molecular phenotypes at single-cell resolution, can be obtained. This approach provides robust data to support the evaluation of the efficacy of MSC therapies. Furthermore, with the advancement of multimodality imaging techniques, it is essential to develop nanoprobes with multiple imaging properties to enable effective imaging of MSCs after transplantation. This is crucial for the clinical tracking and application of MSCs.

## Druggability research for clinical translation

**Fig. 4:**
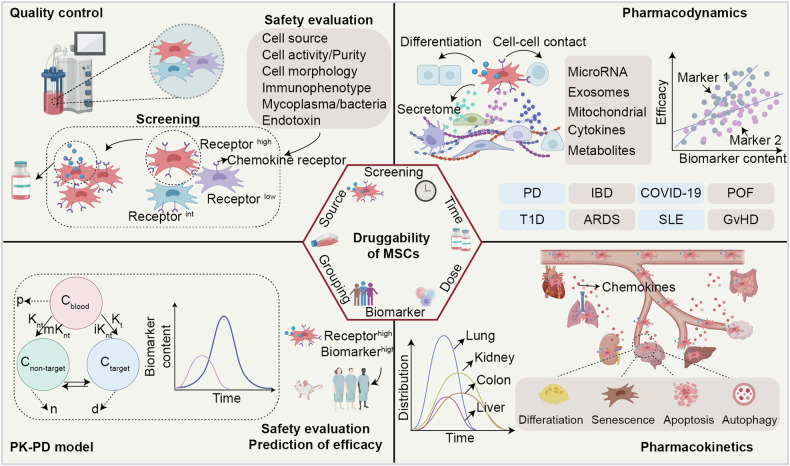
Druggability of MSCs. Applying the right treatment to the appropriate population, in the correct dosage regimen, and at the appropriate time is the most crucial principle in the druggability process of MSCs. The biodistribution and fate of MSCs at different time points were monitored by various imaging technologies. These parameters were combined with pharmacodynamic data to construct a PK/PD model of MSCs. Prediction of the pharmacokinetic properties of MSCs in patients, appropriate dosage regimens, and individualized treatments can be achieved with the PK/PD model. Therefore, the most crucial step in the druggability of MSCs is constructing an appropriate treatment regimen based on the pharmacokinetic and pharmacodynamic properties of MSCs

Like conventional drugs, MSCs also adhere to the guidelines of safety, effectiveness, and quality controllability. Despite their remarkable efficacy in preclinical animal trials, MSC therapies have faced challenges in clinical translation due to individual variability and the considerable number of non-responsive patients.^300^ Several factors may lead to suboptimal clinical outcomes, including heterogeneity in MSC product potency, pharmacokinetics across different administration routes and a limited understanding of how host responses post-administration impact therapeutic efficacy.^301^ Elucidating the relationships among the disease state, biological distribution, and efficacy of MSCs in vivo helps address a significant challenge to their clinical application. Preclinical research on the druggability of MSCs should not only clarify the relationship between their dynamic biodistribution and efficacy but also elucidate their mechanisms of action and develop reliable biomarkers. These efforts will improve patient stratification and facilitate the establishment of precise therapeutic strategies, ultimately optimizing clinical efficacy (Fig. 4).

### Guidelines and consensus

Conventional preclinical and clinical evaluation methods are inadequate for cell-based products due to the differences between cells and conventional pharmaceuticals. The use of MSCs and other cell therapies is under the regulatory oversight of the Center for Biologics Evaluation and Research, specifically the Office of Cellular, Tissue, and Gene Therapies in the United States. MSCs and other cell therapies are categorized under Advanced Therapy Medicinal Products in the European Union. Japan regulates medical products derived from human cells, genes, or tissues under the Pharmaceuticals and Medical Devices Act (PMD Act).^302^ In China, all cell therapies, including MSC therapy products, immune cell products, and gene-modified cell products, can be found in the Technical Guidelines for Research and Evaluation of Cell Therapy Products issued by the NMPA in 2017. The NMPA updated the Biological Products Appendix of the GMP for Drugs in 2020 and drafted the Appendix for Cell Therapy Products in the draft version of the GMP for Drugs. Furthermore, the NMPA drafted the Technical Guidance Principles for Clinical Trials of Human Stem Cells and Their Derived Cell Therapy Products on August 4, 2020. New drug development must adhere to the current Chinese Pharmacopoeia and other national drug standards.^303^ The guidelines and consensus on new drug applications for MSCs or other cell therapy products have been comprehensively reviewed elsewhere.^264,303–305^

### Quality control of MSC therapies

Although the translation of MSCs from bench to bedside is feasible, most MSC therapies face unsuccessful late-stage clinical trials, with notable safety profiles but often limited efficacy in humans. These failures emphasize the challenges associated with the quality control of MSCs for clinical trials.^18^ Given the high heterogeneity and complex biology of MSCs in vivo, enhancing manufacturing processes and formulation technologies and establishing standardized production systems and personalized quality control are essential for successful clinical trials.^301^

The International Society for Cell and Gene Therapy (ISCT) initially established three minimum criteria for human MSCs based on their morphology, surface markers, and trilineage differentiation.^306^ MSCs are defined as adherent cells with a spindle-shaped morphology under standard culture conditions. These cells exhibited the following characteristics: (1) expression of CD105, CD73, and CD90 but lacking expression of CD45, CD34, CD14, or CD11b, CD79a, CD19, or HLA-DR; and (2) the capacity for differentiation into osteoblasts, adipocytes, and chondrocytes in vitro.^307^ These criteria reflected the “stemness” of MSCs rather than determining their therapeutic properties. Given the variations that exist at multiple levels, these criteria were insufficient to define MSCs comprehensively. In 2019, ISCT updated its standards for defining MSCs to include tissue origin and relevant functional assays to clarify their associated therapeutic mode. Furthermore, regulatory agencies mandate the identification, viability, purity, potency, proliferative capacity, genomic stability, and microbiological testing of MSCs.^308,309^

The aforementioned criteria do not encompass the efficacy of MSC therapies; quality control of MSC therapies also includes the following: (1) the source of MSCs, considering the health status, genetics, gender, and age of donors, as well as diverse tissue origins^1,310^; (2) the methods employed for isolating and obtaining cells from these tissues, whether through enzymatic or mechanical dissociation, which can impact the efficacy of MSCs^311^; (3) culture conditions, including aspects such as the composition of the culture medium, oxygen levels, flask/bioreactor, passage number, and cell surface modifications, which may similarly affect the efficacy and homing capacity of MSCs^308,312^; (4) the techniques used for cryopreservation and subsequent thawing/culture rescue, which can influence the viability, function, and homing potential of MSCs^313–315^; and (5) the expression of biomarkers for efficacy and target tissue homing, such as paracrine effectors and chemokine receptors.^316,317^

### Pharmacokinetics of different sources

The biodistribution of MSCs can be influenced by various factors, such as cell type, route of administration, and host immune status. MSCs from different tissues are capable of trilineage differentiation (osteogenesis, chondrogenesis, and adipogenesis) and exhibit similar surface markers, but significant differences still exist in terms of their biological characteristics and functions.^318^ MSCs isolated from BM and adipose tissues express the same surface biomarkers but differ in the expression of cell adhesion molecules such as integrin α4, ICAM-1, CD34, and VCAM-1.^319^ Li et al. compared the proteomes of MSCs derived from the BM, placenta, and umbilical cord to investigate their expression of migration-related proteins, and the results showed that the migration ability of UC-MSCs was weaker than that of MSCs from the BM and placenta (5.9-fold and 3.2-fold, respectively).^320^ However, Hori *et al*. reported that UC-MSCs exhibit greater migration toward lymphocytes than do BM and adipose tissue-derived MSCs.^321^ These differences may be attributed to variations in chemokine receptors among MSCs from distinct tissue sources.

The differences between autologous and allogeneic MSCs extend to their pharmacokinetics. The lower immunogenicity of autologous MSCs enables them to rapidly localize to target tissues or organs without encountering immune rejection. Consequently, they exhibit prolonged survival in the body due to reduced susceptibility to host immune attack and clearance.^322,323^ However, the application of allogeneic MSCs is encouraged because of factors such as their source and quantity. The industry-sponsored production of allogeneic MSCs enables the manufacture of up to 1 million doses per donor for widespread deployment, far exceeding the capacity of autologous MSCs,^324^ indicating that this choice is not solely driven by biological advantages.

### Preclinical pharmacokinetic research

Pharmacokinetic studies of MSC therapy, which track the biodistribution and fate of cells, play an indispensable role in elucidating the in vivo processes and associated biological actions, as well as in explaining the safety and efficacy of cell therapy products. The pharmacokinetic studies also evaluated the dose concentration, interval and route of administration. *The Technical Guidelines for Research and Evaluation of Cell Therapy Products* (China) emphasize the application of one or more methods to monitor the dynamic distribution, migration, homing, survival, and extinction of cell therapy products. Additionally, preclinical pharmacokinetic research should focus on the physiological processes of MSC therapy products in vivo, examining their differentiation capabilities and the biological effects related to their biodistribution.^303,305^ Although none of the agencies recommend methods for analyzing the spatial and temporal distribution of cells, a fundamental principle is that the methods employed should utilize the highest sensitivity currently available. Examples of such methods listed in the guidelines include MRI, PET, SPECT, FLI, autoradiography, PCR, IHC, and in situ hybridization.^264^ Moreover, the selection of appropriate animal models should align with the pathogenesis and therapeutic mechanism of MSCs.^264^ In addition to examining the conventional distribution and migration of MSCs, it is important to consider the impact of gene modification or bioengineering approaches on MSCs, their expression or secretion of biomolecules, and their interactions with host cells, which involve the tissue responses induced by the secretion of bioactive molecules from MSCs.^304^ The preclinical pharmacokinetic characteristics of MSCs can help inform experimental design and patient stratification in clinical pharmacokinetic research.

### Clinical pharmacokinetic research

Clinical pharmacokinetic research cannot employ invasive detection methods such as those used in animal experiments. Extrapolating the results of animal studies of MSC therapies to humans is challenging. The mismatch between mouse and human clinical outcomes may arise from differences in immune compatibility, dosage, condition of culture-adapted MSCs, and variations in disease states of the hosts.^18^ In addition to the aforementioned factors in preclinical pharmacokinetic research, individual variability presents a significant challenge in clinical pharmacokinetic research. The diverse pathological conditions, such as varying levels of inflammation and oxygen, present in clinical subjects greatly impact the pharmacokinetic characteristics of MSCs in the body, thereby influencing the quantity and viability of MSCs that reach specific target organs or tissues.^256^ Moreover, the interactions between MSCs and host cells lead to variations in their therapeutic efficacy.^325^ Alsasem et al. developed an advanced, real-time framework for managing MSC transfusions in COVID-19 patients by automatically categorizing patients into various emergency levels and prioritizing them based on their individual status within each level.^326^ Another important consideration is that the biodistribution of administered MSCs may be influenced by the age of the recipient, which is closely linked to varying metabolic disorders and immunity.^327^ The pharmacokinetic biomarkers of MSCs identified in preclinical studies can be used to predict and validate clinical pharmacokinetics in patients. The development of therapeutic strategies for individual patients to achieve precise and personalized therapy is emerging as a crucial direction for the future advancement of MSC therapy.

### Pharmacokinetics model for systemic administration

To satisfy the clinical requirement for the accuracy of MSC dosage, several pharmacokinetic models have been designed. Previous studies have used a two-compartment pharmacokinetic model, referring to MSCs as inactive and micron-sized nanoparticles, to predict their distribution, yielding remarkably similar data to the actual bioavailability of MSCs post-transplantation.^328^ However, this model was unable to predict the precise biodistribution of MSCs due to its disregard of MSCs as a dynamic “living therapy”. By integrating anatomical structure into the design, the physiologically based pharmacokinetic (PBPK) model offers greater physiological similarity and prediction accuracy. The first PBPK model of MSCs, reported in 2016, used in vivo optical imaging to track MSC biodistribution and flow cytometry to quantify MSCs.^329^

Chimeric antigen receptor (CAR)-T cells are recognized as effective therapeutic agents for antitumour applications. Given the similarities between CAR-T cells and MSCs in terms of therapeutic mechanism and cell properties, the model structure and system-specific parameters of the CAR-T-cell PK model can be applied to MSCs. PBPK models of CAR-T cells have been developed that incorporate various parameters, such as tissue volume, blood flow velocity, affinity of CAR-T cells for target cells, antigen abundance, and tumor volume.^330^ To accurately predict the biodistribution of CAR-T cells in vivo, the types of target cells and the expression of ligands on target cells in different disease stages, which could represent the specific properties of CAR-T cells, should be considered in the development and optimization of models.^331^ The target organ volume, microenvironment, and interaction between MSCs and other cells can all be considered important parameters or processes for PK models. Furthermore, the physical status of donors, tissue resources, passage number of MSCs, administration route, and dose of MSCs can all be recognized as critical covariates in models because of their unignorable influence on the biodistribution of MSCs.^332^ To maximize the therapeutic effects and minimize the adverse effects of MSCs, a PK model that has a superior ability to predict the biodistribution and number of MSCs in target organs is urgently needed.

## Conclusions

MSC therapy, which has undergone significant progress over the past half-century, has emerged as an effective treatment for major chronic diseases and severe trauma repair in the field of regenerative medicine.^27^ Although the safety and therapeutic efficacy of MSC therapy have been extensively validated in numerous clinical trials worldwide, only a limited number of MSC products are commercially available in specific regions. This is largely attributed to the challenges associated with pharmacokinetics and the lack of clarity regarding MSC mechanisms, as well as the substantial individualized differences observed in clinical trials. In this review, we discuss MSC homing processes to different organs in vivo and the key regulatory factors involved. We also analyzed MSC fate and evaluated various in vivo tracing methods for detecting MSC homing, highlighting their advantages and disadvantages. Furthermore, we have emphasized the close relationship between the pharmacokinetics of MSCs and their druggability. We have summarized current strategies and suggestions for enhancing the homing capabilities of transplanted MSCs.

MSC therapy products are not subject to conventional pharmacokinetic testing, which is primarily used for conventional drugs. The mode of action of MSCs operates through a “shotgun model” and “hit-and-run” (or “touch-and-go”) mechanism: (1) They exert regulatory effects on the body through multiple targets and mechanisms to maintain homeostasis within niches rather than through single-target and simple inhibitory actions^9^; (2) Many studies have shown their rapid migration to damaged tissue, where they are quickly cleared after stress-induced release of therapeutic molecules, but their therapeutic effects persist over a long period.^12^ However, there are still many unanswered questions. First, given the high heterogeneity of MSCs, future research should focus on homing studies for different indications to further standardize quality criteria and production processes, ensuring the consistency of MSC product batches. Second, most studies have not linked the fate of MSCs to persistence in vivo due to limitations in detection and labeling technologies. Combining multiple labeling methods and various imaging techniques will facilitate high-resolution imaging and quantitative spatiotemporal analysis of MSCs in target organs at the single-cell resolution level. Third, it is important to note that preclinical mouse models often do not fully replicate human diseases. Although MSCs exhibit very low immunogenicity, they can trigger varying levels of immune activation in both humans and animals. Additionally, the dosage of MSCs administered to mouse models differs significantly from that administered to clinical patients, which can affect treatment outcomes. Finally, screening response patients based on biomarkers to determine the biodistribution and biological mechanisms of MSCs before clinical application can enhance the efficacy of MSC therapies. On the other hand, combining MSCs with gene modification, click chemistry, and tissue engineering materials hold promise for enhancing the precise biodistribution of MSC therapies. This innovative approach has the potential to accelerate the clinical translation of MSCs and expand their application to new therapeutic frontiers, ultimately benefiting patients with a wide range of medical conditions.
